# Prospective biomarkers of major depressive disorder: a systematic review and meta-analysis

**DOI:** 10.1038/s41380-019-0585-z

**Published:** 2019-11-19

**Authors:** Mitzy Kennis, Lotte Gerritsen, Marije van Dalen, Alishia Williams, Pim Cuijpers, Claudi Bockting

**Affiliations:** 1 Department of Clinical Psychology, Utrecht University, Utrecht, The Netherlands; 2 School of Psychology, Faculty of Science, the University of New South Wales, Sydney, NSW Australia; 3 Department of Clinical, Neuro and Developmental Psychology, Amsterdam Public Health research institute, Vrije Universiteit Amsterdam, Amsterdam, The Netherlands; 4 Department of Psychiatry, Amsterdam University Medical Centers, location AMC, University of Amsterdam, Amsterdam, The Netherlands; 5 Institute for Advanced Study, University of Amsterdam, Amsterdam, The Netherlands

**Keywords:** Prognostic markers, Psychology, Neuroscience

## Abstract

Leading biological hypotheses propose that biological changes may underlie major depressive disorder onset and relapse/recurrence. Here, we investigate if there is prospective evidence for biomarkers derived from leading theories. We focus on neuroimaging, gastrointestinal factors, immunology, neurotrophic factors, neurotransmitters, hormones, and oxidative stress. Searches were performed in Pubmed, Embase and PsychInfo for articles published up to 06/2019. References and citations of included articles were screened to identify additional articles. Inclusion criteria were having an MDD diagnosis as outcome, a biomarker as predictor, and prospective design search terms were formulated accordingly. PRISMA guidelines were applied. Meta-analyses were performed using a random effect model when three or more comparable studies were identified, using a random effect model. Our search resulted in 67,464 articles, of which 75 prospective articles were identified on: Neuroimaging (*N* = 24), Gastrointestinal factors (*N* = 1), Immunology (*N* = 8), Neurotrophic (*N* = 2), Neurotransmitters (*N* = 1), Hormones (*N* = 39), Oxidative stress (*N* = 1). Meta-analyses on brain volumes and immunology markers were not significant. Only cortisol (*N* = 19, OR = 1.294, *p* = 0.024) showed a predictive effect on onset/relapse/recurrence of MDD, but not on time until MDD onset/relapse/recurrence. However, this effect disappeared when studies including participants with a baseline clinical diagnosis were removed from the analyses. Other studies were too heterogeneous to compare. Thus, there is a lack of evidence for leading biological theories for onset and maintenance of depression. Only cortisol was identified as potential predictor for MDD, but results are influenced by the disease state. High-quality (prospective) studies on MDD are needed to disentangle the etiology and maintenance of MDD.

## Introduction

Major depressive disorder (MDD) is a disabling disorder that is amongst the most prevalent mental health disorders worldwide [1, 2] and is highly recurrent [3–5]. Therapeutic strategies, such as antidepressant medication, are available, although outcomes are suboptimal given roughly 50% of patients do not adequately respond [6, 7]. In order to improve treatment approaches and prevent recurrence, it is important to examine the underlying vulnerabilities that predispose individuals to depression onset and recurrence. By prospectively investigating biological predictors of MDD onset, relapse and recurrence, more insights into the potential causes of MDD can be gained. For these purposes, biomarkers could be particularly informative for understanding the etiology of MDD, and could stimulate development of new clinical approaches in the future.

Numerous studies suggest that MDD is related to alterations in various biological systems [8, 9]. For instance, MDD has been associated with alterations in brain structure and function, (e.g. [10, 11]), gastrointestinal factors (e.g. [12, 13]), immunology (e.g. [14]), endocrinology (including neurotransmitters, e.g. [15, 16]), neurotrophic factors (e.g. [17, 18]), hormones (e.g. [19]), and oxidative stress (e.g. [20]). Based on these frequently reported biomarker alterations several biological hypotheses for the etiology of MDD have been formulated. Support for these hypotheses have primarily been derived from cross-sectional studies. However, cross-sectional studies cannot provide evidence for *causality*, and thus cannot distinguish causes from consequences secondarily to the illness [21]. To determine whether an etiological mechanism is potentially causal for the development of MDD, the minimal requirement for a study is that the biomarkers are assessed before the development of MDD or prior to a recurrent episode. Thus, prospective studies investigating biomarkers before the onset or relapse/recurrence of MDD are necessary. Further, there are indications that first onset versus relapse/recurrence of MDD is based on different mechanisms [22, 23]. Therefore, investigating predictive biomarkers for onset and relapse/recurrence separately can improve predictive models. However, to our knowledge, no systematic overview of prospective studies comparing biomarkers of onset and relapse/recurrence of MDD has been conducted.

Therefore, we will provide a systematic overview of prospective studies investigating leading biological hypotheses on the etiology of MDD. The first goal is to determine whether there is prospective evidence that these biomarkers predict onset, and relapse/recurrence of MDD. A systematic search for prospective studies will be performed. We explicitly focus on studies using a clinical interview to determine the onset and re-occurrence of a major depressive episode. The search is subdivided into the following biological areas: neuroimaging, gastrointestinal, immunology, neurotrophic, neurotransmitters, hormones, and oxidative stress (see Supplementary Fig. 1). The second goal will be to establish the robustness of each biomarker and to compare the effect size of different biomarkers. Further, subgroup analyses and meta-regression will be performed to investigate potential moderators.

## Methods

### Search process and study selection

The study was performed according to Preferred Reporting Items for Systematic Reviews and Meta-Analyses statement (PRISMA [24]; see Appendices A and B for search terms and flow charts and Appendix C for PRISMA checklist). This meta-analysis was part of a larger project on evidence for leading theories for MDD onset, and relapse/recurrence and mechanisms of change (for the current study see registration in Prospero CRD42017072990; for psychological predictors of depression see Prospero CRD42017073975; CRD42017073977). Literature searches per biological system were performed between July 2016 and July 2017 in the online databases PubMed, PsychINFO and EMBASE, and a combined search update was performed in June 2019. No start date was included, so all articles that were digitalized until June 2019 were included. The search included terms related to: (1) MDD, (2) longitudinal studies predicting onset, relapse and recurrence, and (3) biological systems of interest (see Appendix A). The articles were independently screened for eligibility based on title and abstract (see criteria below) by two team members, including at least one of the researchers (MK, LG, or MvD), and a member of our screening team (psychology/research Master students; see “Acknowledgements”).

The following inclusion criteria were applied: (1) Diagnostic status of MDD for all participants through clinical interview (e.g., SCID, K-SADS from DSM, CIDI from ICD) or report of a clinician-assessed diagnosis (e.g., being hospitalized for MDD treatment, self-report of being diagnosed with MDD by a clinician). (2) The study design is longitudinal. (3) The target variable(s) (e.g., the proposed vulnerability factors) are assessed prospectively, that is before the onset or relapse/recurrence of MDD. (4) The target variable is derived from one of the leading biological models. Exclusion criteria were: diagnosis of mood disorders other than MDD (e.g. bipolar disorder), late-life depression, MDD due to the other (medical) disorders, or studies including a mixed group of diagnoses where less than 75% was diagnosed with MDD. In order to trace studies published after the initial search date, and to add recently published studies, we screened of the included articles the reference list, articles citing, and reference lists of recent reviews. This was done between August and September 2017, and in June 2019 for the new inclusions.

### Data extraction and quality assessment

Data extraction was performed by two team members independently (but not blind to the data extracted by the first data extractor) including at least one author (MK, LG, and MvD) and a member of our screening team (see “Acknowledgement”). The following data were extracted: number of included participants and group membership (developing MDD or not), age, gender, study country, MDD diagnosis at baseline, assessment tool of diagnosis, diagnostic criteria, biomarker measurement outcome, biomarker type of measurement, biomarker time of measurement, follow-up time, summary of main outcome. The quality of included studies was assessed by two team members according to a minimally adjusted version of the GRADE guidelines on study level [25]. Information was extracted on selection of cohorts (similar for groups compared), quality of MDD assessment instrument, presence of baseline MDD (symptoms), matching of samples or adjustion for covariates, biomarker assessment, interviewer, description of drop-outs, description of interventions, and other sources of bias. A score for the quality was also given, by counting the number of questions where there was limited risk of bias (max score = 9).

### Analysis

Random effects meta-analyses were performed using comprehensive meta-analysis (www.meta-analysis.com). A meta-analysis was conducted when three or more studies were included using a similar modality of biomarker assessment [26]. When multiple studies investigated the same sample, analysis included only the study with the largest sample size. Odds ratio or risk ratio were the summary effects of outcome. Significance was determined with *p* = 0.05 for meta-analyses. First, analysis was performed on onset and relapse/recurrence of MDD combined to investigate the predictive effect of all biomarkers on MDD development in general. Differences between biomarker effects was also investigated with a subgroup analysis. If a difference exists, meta-analyses were performed per biomarker. Second, separate analyses were performed on studies including participants without baseline clinical MDD diagnosis and/or first onset only versus studies including participants with baseline clinical MDD diagnosis and/or relapse/recurrence (including mixed groups with onset and relapse/recurrence). Heterogeneity was assessed with the *Q*-test and *I*^2^ statistic [27]. Sensitivity analyses were also performed by re-running analyses after removal of outliers (defined by having no overlap of the 95% CI with the pooled effect 95% CI) and studies with low risk of bias. Baseline age, percentage female participants, biomarker assessment, follow-up time, and quality assessment score were assessed as moderators, when sufficient studies (three per subcategory) were included in the analysis. For analysis of biomarker assessment all effect sizes reported were taken into account. Publication bias was also assessed using Egger’s test for asymmetry [28] of the funnel plot and Duval and Tweedie’s trim and fill procedure [29].

## Results

### Search results and quality assessment

**Fig. 1:**
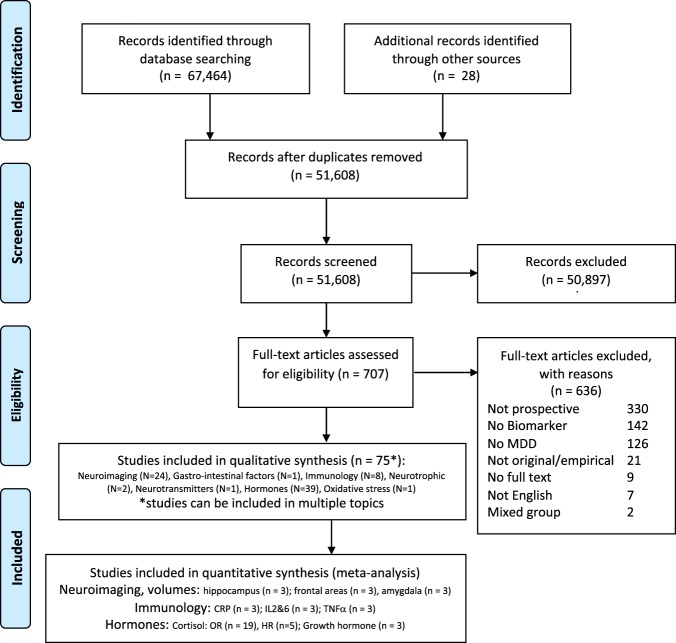
Flow Diagram of systematic search for prospective studies of MDD overall biological searches combined [24]. See Supplementary material for flow charts per search

The PRISMA flow chart provides an overview of the number of articles screened, included and excluded for all biomarkers combined (see Fig. 1; flow charts per biological system can be found in Appendix B). In total, 67,464 articles were screened for eligibility across all biomarkers.^1^ After initial screening, eligibility of 707 articles was assessed based on the full text. In total, only 75 unique prospective studies were identified (see Table 1; [30–104]). Overall, 75 prospective articles were identified on: Neuroimaging (*N* = 24), Gastrointestinal factors (*N* = 1), Immunology/inflammation (*N* = 8), Neurotrophic (*N* = 2), Neurotransmitters (*N* = 1), Hormones (*N* = 39), and Oxidative stress (*N* = 1). In total 39,028,432 participants (median 85, range [9–9275]) were included (Table 1), of which 3267 developed MDD over the follow-up period (median 22, range [3–608]). The median age of study participants was 39 [range 9–66] and the the median percentage of females included was 64% [29–100%]. Follow-up time ranged from 4 months to 22 years, which is adequate for detecting onset, relapse or recurrence (median 3 years). The SCID (*N* = 23) and versions of the (K)SADS (*N* = 19) were the most frequently administered clinical interviews to assess MDD using DSM criteria (DSM *N* = 54) over ICD criteria. Studies describing a clinical diagnosis made by two independent psychiatrist or self-report of hospitalization or diagnosis for MDD were also included incidentally (*N* = 7). Most studies were performed in Western countries (e.g. USA, UK, and Germany, see Table 1). Only 38 studies were identified that excluded participants with baseline clinical MDD diagnosis. First onset of MDD was investigated in 31 studies, relapse/recurrence in 35 studies, and 9 studies included mixed onset and relapse/recurrence samples. Overall, the mean quality score of studies was good (average quality score = 6.3, median 6, range (3–9)), 19 studies had a very low risk of bias (>6 quality score), 26 studies had some risk of bias (5–6 quality score), and 8 studies had high risk of bias (4 or lower quality score). Below, meta-analyses will be described and incidental findings will be discussed narratively (see tables in Supplementary material).

**Table 1:** Study details on all prospective studies (*N* = 75), subdivided by the following biological sublevels: Neuroimaging, Gastrointestinal factors, Immunology, Neurotransmitters, Neurotrophic factors, Hormones: HPA axis, HPG axis, HPS axis, and HPT axis

Neuroimaging	Baseline MDD excl. Y = yes, N = No	Baseline symptoms a = above cut-off, b = below cut-off, ? = unclear	Country and cohort information (nr indicates similar cohorts)	Total *N*	Onset (O) or relapse/ recurrence (R) of MDD N	Baseline age (mean or range)	% female	Follow-up time (years; mean or max)	MDD diagnostic interview; Diagnostic criteria	Biomarker of interest	Measure	Technical details	Direction result of biomarker predicting onset, relapse/ recurrence of MDD	Quality score
Bress et al. [39]^a^	Y	b	USA	68	16 O	[15–17]	100	2	DISC; ICD-10	Frontal brain areas (ERP)	Activity: EEG	During reward task. comparing loss-gain	↓ Frontal ERP	7
Davey et al. [49]^a^	Y	?	Australia	56	8 O	17	45	2	K-SADS-PL; DSM-IV (nm)	Amygdala-sgACC connectivity	RSFC fMRI	Resting state	↑ Amygdala–sACC connectivity	4
Foland-Ross et al. [51]^b^	Y	b	USA	33	18 O	13	100	5	K-SADS-PL and SCID; DSM-IV (nm)	32 brain regions were used for support vector machine classification	Structure: MRI	Volumes	The most important classifiers for MDD were ↓ mOFC, PCG, AC*C*, and ↑ insula	7
Little et al. [73]^c^	Y	?	Australia1	99	26 O	13	29	6	K-SADS-PL. MINI; DSM-IV (nm)	Hippocampus, amygdala, OFC and ACC	Structure: MRI	Volumes	↓ Hippocampus NS amygdala, OFC and ACC	6
Little et al. [74]	?	?	Australia1	137	36 O	13	52	6	K-SADS-PL; DSM-IV (nm)	Hippocampus	Structure: MRI	Volumes	↓ hippocampus	5
Nusslock et al. [83]^a^	?	b	USA	40	13 O	20	43	3	SADS-C; DSM-IV	Left frontal brain areas (Alpha power)	Activity: EEG	Rest. eyes-open and eyes-closed	↓ Left frontal activity	6
Papmeyer et al. [85]^a^	Y	b	UK1	204	19 O	21	56	2	Clinical interview; DSM-IV (nm)	Frontal brain regions	Structure: MRI	Gray matter thickness	↓ Right parahippocampal and fusiform gyrus	9
Papmeyer et al. [86]^a^	Y	b	UK1	204	19 O	21	56	2	Clinical interview; DSM-IV	Subcortical brain regions	Structure: MRI	Gray matter thickness	NS	9
Whalley et al. [99]^a^	Y	b	UK1	156	20 O	21	52	2	SCID ; DSM-IV	Brain regions activated by the task	Activity: fMRI	Cognitive sentence completion task: increase in difficulty	↑ Insula activity	7
Whalley et al. [100]^a^	Y	b	UK1	50	11 O	23	Unclear	2	SCID; DSM-IV	Amygdala, insula, hippocampus, ACC, thalamus	Activity: fMRI	View emotional images vs neutral	↑ Thalamus, insula, ACC activity	7
Nickson et al. [81]^c^	N	b	UK1	131	30 O	21	69	4.9	SCID; DSM-IV	VBM	Structure: MRI	Whole brain VBM	↑Amygdala gray msatter volume	7
Macoveanu et al. [77]^b^	Y	b	Denmark	85	12 O	39	65	7	SCAN 2.1; (ICD-10)	VBM	Structure: MRI	Whole brain VBM	↑ACC gray matter volume	5
Belden et al. [36]^a^	Y	?	USA	129	24 O + R	[6–12]	48	10	Clinician and TRD; DSM-5	Anterior insula	Structure: MRI	Volumes	↓ Insula volume	6
Rao et al. [91]^a^	Y	a	USA	83	29 O + R	15	58	5	LIFE. PSR; DSM-IV (at baseline)	Hippocampus	Structure: MRI	Volumes	↓ Hippocampus	5
Serra-Blasco et al. [95]^a^	N	a	Spain	24	10 O + R	48	75	5	Life-Chart Manual for Recurrent Affective Illness; Unclear	Whole brain (Freesurfer)	Structure: MRI	Volumes	↓rIFG, ACC, rMFG volumes predict recurrence	8
Allen et al. [31]^a^	Y	b	USA	9	3 R	49	61	0.5	SCID; DSM-III-R	Frontal asymmetry	Activity: EEG	Rest. pre- post-tryptophan depletion	↑ Right frontal activity after vs before TD predict lower change of recurrence at follow-up	6
Farb et al. [50]^a^	Y	b	Canada	16	10 R	39	69	1.5	SCID; DSM-IV	ROI mPFC	Activity: fMRI	Viewing sad vs natural movie clips	↑ mPFC activity	7
Frodl et al. [53]^c^	N	a	Germany1	30	12 R	48	60	1.5	Clinical interview, 2 psychiatrists; DSM-IV	Hippocampus and amygdala	Structure: MRI	Volumes	↓ Hippocampus NS amygdala	6
Frodl et al. [54]	N	a	Germany1	30	13 R	45	63	3	SCID; DSM-IV	Hippocampus and amygdala	Structure: MRI	Volumes	NS	6
Kronmüller et al. [70]^b^	N	a	Germany	57	21 R	44	58	2	SCID / LIFE; DSM-IV	Hippocampus	Structure: MRI	Volumes	↓ Hippocampus	5
Lythe et al. [76]^a^	Y	b	UK	95	25 R	34	64	1.2	LIFE; DSM-IV	ROI based on previous studies: ACC, temporal, striatal	Activity: fMRI	Activity during self-blame versus other-blame emotions	↑ sgACC and temporal regions connectivity and putamen and claustrum connectivity in recurrent	9
Nixon et al. [117]^a^	Y	b	UK	38	7 R	[24–63]	33	1	SCID; DSM-IV	ROI based on previous studies	Activity: fMRI	Go/Nogo task	↓ Right dmPFC activity following errors and negative feedback compared to correct hits in recurrence vs other groups	6
Workman et al. [101]^a^	Y	?	UK	85	17 R	37	64	1.2	SCID; DSM-IV	sgACC	RSFC: fMRI	Rest. Left sgACC to right sgACC connectivity	Recurrent group was intermediate to resilient and control in sgACC connectivity	9
Langenecker et al. [71]^a^	Y	b	USA	94	21 R	21	63	4–6	DIGS and longitudinal interval follow evaluation	fMRI	Activity: fMRI & RSFC fMRI	Go/No-Go task: successful vs unsuccessful inhibition	Successful regulation sgACC hyperactivation. Failed regulation; MFG hypoactivation. RSFC: altered MFG and sgACC connectivity.	8
**Gastrointestinal factors**	**Baseline MDD excl. Y** **=** **yes, N** **=** **No**	**Baseline symptoms a** **=** **above cut-off, b** **=** **below cut-off, ?** **=** **unclear**	**Country and cohort information (nr indicates similar cohorts)**	**Total** ***N***	**Onset (O) or relapse/ recurrence (R) of MDD (N)**	**Baseline age (mean or range)**	**% female**	**Follow-up time (years; mean or max)**	**MDD diagnostic interview; Diagnostic criteria**	**Biomarker of interest**	**Measure**	**Technical details**	**Direction result of biomarker predicting onset, relapse/recurrence of MDD**	**QA score**
Campo et al. [40] °	Y	?	USA	119	14 O	[6–12]	?	7.5	K-SADS-E; DSM-IV (nm)	L-5-hydroxytryptophan injection at baseline	Abdominal discomfort, nausea, or vomiting in response to the L-5HTP infusion; GI distress	L-5HTP infusion; Survival curve (groups high vs. low sensitive)	↑GI distress after serotonin challenge	6
**Immunology**	**Baseline MDD excl. Y** **=** **yes, N** **=** **No**	**Baseline symptoms a** **=** **above cut-off, b** **=** **below cut-off, ?** **=** **unclear**	**Country and cohort information (nr indicates similar cohorts)**	**Total** ***N***	**Onset (O) or relapse/ recurrence (R) of MDD (N)**	**Baseline age (mean or range)**	**% female**	**Follow-up time (years; mean or max)**	**MDD diagnostic interview; Diagnostic criteria**	**Biomarker of interest**	**Measure**	**Technical details**	**Direction result of biomarker predicting onset, relapse/ recurrence of MDD**	**QA score**
Chocano-Bedoya et al. [43]^c^	N	?	USA	4403	81 O	56	100	12	Self-report of diagnosis; Unclear	CRP, IL-6, TNFα-R2	Blood, 1 time point	hs-CRP IA. EIA	NS	4
Haastrup et al. [61]^a^	N	?	Denmark	9275	22 O	[18–47]	48	5	prescriptions for antidepressant medication or had a hospital discharge diagnose with the codes F.32 or F.33; ICD-10	suPAR	Blood plasma, 1 time point	ELISA	↑Increased risk (shorter time) to onset of depression with higher SuPAR	6
Rudaz et al. [94]	Y	?	Switzerland1	1524	192 O	51	43	5.5	Diagnostic Interview for Genetic Studies (DIGS); DSM-IV	CRP, IL-1ß, IL-6, TNFα	Blood serum, 1 time point	hs-CRP IA. multiplexed particle-based flow cytometric cytokine assay	↓TNFα, NS for CRP, IL-1ß, IL-6	8
Copeland et al. [46]^c^	Y	b	USA	5810	169 O + R	14	49	12	CAPA, YAPA; DSM-IV	CRP	Blood spot, 1 time point	hs-CRP IA	NS	9
Glaus et al. [55]^c^	Y/N	?	Switzerland1	2580	608 O + R	[35–66]	54	5.5	DIGS	CRP, IL-6, TNFα	Serum, 1 time pont	IA and latex HS	O + R: ↓TNFα	7
Khandaker et al. [69]^c^	N	?	UK	2447	422 O + R	9	51	9	CIS-R; ICD-10	CRP, IL-6	Blood serum, 1 time point	hs-CRP essay. ELISA	NS	5
Pasco et al. [87]^a^	Y	?	Australia	822	151 O + R	49	100	10	SCID; DSM-IV-TR	CRP	Blood serum, 1 time point	hs-CRP IA	↑ CRP reduced time till depression (HR)	7
**Neurotransmitters**	**Baseline MDD excl. Y** **=** **yes, N** **=** **No**	**Baseline symptoms a** **=** **above cut-off, b** **=** **below cut-off, ?** **=** **unclear**	**Country and cohort information (nr indicates similar cohorts)**	**Total** ***N***	**Onset (O) or relapse/ recurrence (R) of MDD (N)**	**Baseline age (mean or range)**	**% female**	**Follow-up time (years; mean or max)**	**MDD diagnostic interview; Diagnostic criteria**	**Biomarker of interest**	**Measure**	**Technical details**	**Direction result of biomarker predicting onset, relapse/ recurrence of MDD**	**QA score**
Johnston et al. [68]^a^	N	a	UK	31	20 R	47	71	8	SCID I/P; DSM-III-R	Plasma Norepinephrine, cortisol	Blood plasma, 1 time point	Chromatography, RIA	↓ Norepinephrine, shorter time to first recurrence.	6
**Neurotrophic factors and oxidative stress**	**Baseline MDD excl. Y** **=** **yes, N** **=** **No**	**Baseline symptoms a** **=** **above cut-off, b** **=** **below cut-off, ?** **=** **unclear**	**Country and cohort information (nr indicates similar cohorts)**	**Total** ***N***	**Onset (O) or relapse/ recurrence (R) of MDD (N)**	**Baseline age (mean or range)**	**% female**	**Follow-up time (years; mean or max)**	**MDD diagnostic interview; Diagnostic criteria**	**Biomarker of interest**	**Measure**	**Technical details**	**Direction result of biomarker predicting onset, relapse/recurrence of MDD**	**QA score**
Pasquali et al. [88]^a^	N	b	USA	148	37 O	40	100	3	SCID; DSM-IV	*Neurotrophic*: BDNF *Immunology:* HSP70, 3-Nitrotyrosine,, *Oxidative stress:* Protein carbonyl, Lipid peroxidation, Thiol content	Blood serum, 1 time point	ELISA, quantitative sandwich enzyme immunoassay, colorimetric assay (thiol)	↑ heat-shock protein 70, 3-nitrotyrosine, protein carbonyl, and lipid peroxidation ↓BDNF	6
Vinberg et al. [97]^a^	Y	?	Denmark	234	24 O	44	65	7.5	SCAN; ICD 8/ICD-10	BDNF	Blood, 1 time point	Two-site sandwich ELISA	NS	5
**Hormones: HPA**	**Baseline MDD excl. Y** **=** **yes, N** **=** **No**	**Baseline symptoms a** **=** **above cut-off, b** **=** **below cut-off, ?** **=** **unclear**	**Country and cohort information (nr indicates similar cohorts)**	**Total** ***N***	**Onset (O) or relapse/ recurrence (R) of MDD (N)**	**Baseline age (mean or range)**	**% female**	**Follow-up time (years; mean or max)**	**MDD diagnostic interview; Diagnostic criteria**	**Biomarker of interest**	**Measure**	**Technical details**	**Direction result of biomarker predicting onset, relapse/ recurrence of MDD**	**QA score**
Adam et al. [30]^c^	N	?	USA	230	18 O	17	75	1	SCID; DSM-IV	AUC, CAR, diurnal, slope	Saliva, 3 days, 6 times a day	DELFIA	NS	7
Colich et al. [45]^c^	Y	b	USA1	89	31 O	12	100	6	K-SADS. SCID; DSM-IV	Cortisol pre- post- trier social stress test	Saliva, 4 time points	LIA	↓ cortisol reactivity in early puberty ↑cortisol reactivity in late puberty	9
Goodyer et al. [56]	?	?	UK2	171	30 O	14	59	1	K-SADS; DSM-IV	Peaks 8:00, cortisol and DHEA	Saliva 4 days, 2 time points	ELISA	↑ cortisol & DHEA	6
Goodyer et al. [57]	?	?	UK2	234	31 O	14	53	1	K-SADS; DSM-IV	Morning and evening cortisol, DHEA	Saliva 4 days, 2 time points	ELISA	↑ DHEA, NS cortisol	7
Goodyer et al. [58]^c^	?	b	UK2	367	32 O	14	46	1	K-SADS; DSM-IV	Morning cortisol	Saliva 4 days, 1 time point	ELISA	↑ cortisol	6
Goodyer et al. [59]	?	b	UK2	357	40 O	14	47	1	K-SADS; DSM-IV	Morning cortisol	Saliva 4 days, 1 time point	ELISA	↑ cortisol	6
Grynderup et al. [60]^c^	Y	?	Denmark	2920	62 O	56	78	2	SCAN; ICD-10-DSR	Morning, diurnal, evening, morning to evening slope in cortisol	Saliva, 2 hours after awakening, and between 5PM and 4AM	RIA	↓ difference in morning to evening cortisol	9
Harris et al. [63]^c^	?	?	UK	116	28 O	39	100	1	SCAN; DSM-IV	DHEA and cortisol	Saliva, 4 days, 2 time points	ELISA	NS	6
Herbert et al. [65]^c^	Y	?	UK	279	53 O	37	100	1	SCAN; DSM-IV	Morning cortisol	Saliva 4 days, 1 time point	ELISA	↑ cortisol predicts MDD	8
LeMoult et al. [72]	Y	b	USA1	62	26 O	12	100	6.5	K-SADS. SCID; DSM-IV	Morning cortisol	Saliva, 2 days, 4 time points	ELISA	↑ cortisol predicts MDD	9
Rao et al. [93]^c^	Y	b	USA2	89	14 O	15	58	5	K-SADS; DSM-IV	Saliva, NUFC	Saliva, 3 time points before sleep and Urine 1 time point before sleep	RIA	↑ cortisol predicts MDD	6
Carnegie et al. [41]^c^	N	?	UK	841	46 O + R	15	49	3	CIS-R; ICD-10	AUC, CAR, DD, bedtime (m), waking (m), TEC	Urine, over 24 h	EIA	NS	5
Vrshek-Schallhorn et al. [98]^c^	Y	?	USA	270	42 O + R	17	72	4	SCID; DSM-IV	AUC, CAR, diurnal slope	Saliva, 3 days, multiple time points	Time-resolved fluorescent-detection IA	↑ cortisol recurrence	9
Appelhof et al. [32]^c^	N	a	Spain	45	22 R	50	44	22	At baseline: SCID. Relapse: HRSD. MADRS. and BD; DSM-IV	Post dex cortisol, Max ACTH, Delta ACTH, Max cortisol, delta cortisol, discharge, difference cortisol	Blood, 2 days, multiple time points before and after DEX/CRH combined with TRH	luminescen-ce enzyme IA	↑ maximal cortisol after DEX/CRH predicts relapse	6
Aubry et al. [34]^b^	Y	a	Switzerland	34	12 R	44	56	1	MINI; DSM-III-R/IV. ICD-10	Cortisol after DEX/CRH test	Blood, 2 days, multiple time points before and after DEX/CRH	Immulite 2000 analyser	↑ AUC and delta in relapse	8
Banki et al. [35]^b^	N	a	Hungary	24	9 R	51	100	0.5	Hospital diagnosis; DSM-III-R	CRH, SRIF	CSF, 1 time point	Sensitive and specific IA	↑ CRF in relapse	3
Bockting et al. [37]^c^	Y	b	Netherlands1	55	43 R	44	73	5.5	SCID; DSM-IV	Morning and evening cortisol	Saliva, 2 days, 1–2 time points a day	RIA	↓ cortisol related to relapse	7
Bouhuys et al. [38]^c^	N	b	Netherlands	77	21 R	44	66	2	CIDI; DSM-IV	24 h urine		RIA	NS	6
Charles et al. [42]^c^	N	?	Belgium	13	7 R	[33–67]	77	1.5	SADS-L; DSM-III and RDC	Morning after DST	Blood plasma, 2 time points after taking DEX	RIA	↑ cortisol (non suppression at recovery) higher rates of MDD readmission	4
Chopra et al. [44]^b^	Y	b	Canada	55	28 R	39	64	1.5	SCID; DSM-IV	Morning/evening (before mood induction)	Saliva, 4 time points	EIA	↑ cortisol	4
Cosgriff et al. [48]^c^	N	b	New Zealand	13	4 R	51	54	0.25	Clinical readmission; Unclear	Mean cortisol, Delta TSH	Blood, 10–12 time points	EIA/RIA	↑ cortisol	5
Hardeveld et al. [62]^c^	Y	a	Netherlands	549	193 R	45	71	4	CIDI; DSM-IV	Salivary CAR, evening, DST	Saliva, 2 days, 6 times day 1, 1 time day 2 after DEX	EIA	AUC increase differed, and related to time to recurrence. Other measures, mean evening, DST and AUC were not predictive	7
Hatzinger et al. [64]^b^	N	a	Switzerland	20	12 R	52	70	1	SCID; ICD-10	Cortisol after DEX/CRH	Blood plasma, 1 time point after DEX/CRH	RIA	DEX/CRH test	7
Lok et al. [75]	Y	b	Netherlands1	187	102 R	44	68	2	SCID; DSM-IV	Morning and evening cortisol	Saliva, 2 days, 3 time points	RIA	NS	8
Mander et al. [78]^c^	N	?	UK	70	32 R	Unclear	Unclear	3	SCID; DSM-III	DEX suppression	Saliva, 1 day, 3 time points day after DEX	RIA	NS	5
Mocking et al. [118]^c^	Y	b	Netherlands1	187	154 R	44	68	10	SCID; DSM-IV	Cortisol/ DHEAS ratio	Saliva, 2 days, 2 time points a day.	RIA	↓DHEAS diurnal course, ↑ cortisol/DHEAS ratio diurnal course	6
Morris et al. [80]^c^	Y	a/b	USA	32	9 R	23	63	0.75	SCID; DSM-IV	Cortisol pre- post- TSST	Saliva, 6 time points	ELISA	NS	8
Pintor et al. [89]	N	a	Spain1	43	18 R	51	53	2	SCID; DSM-IV	Cortisol and ACTH after CRF injection	Blood plasma, 6 time points around CRF injection	EIA/RIA	NS Cortisol and ACTH AUC, ACTH after CRF	6
Pintor et al. [90]^c^	N	a	Spain1	43	18 R	51	46	2	SCID; DSM-IV	Cortisol and ACTH after CRF injection	Blood plasma, 6 time points around CRF injection	EIA/RIA	↑ cortisol (NAUCC) after CRF, ↓ACTH after CRF	5
Rao et al. [92]	N	b	USA2	47	20 R	15	64	3.5	K-SADS-PL; DSM-IV	NUFC	Urine before and after sleep	RIA	↑ cortisol	5
Tsuru et al. [96]^c^	N	a	Japan	25	9 R	41	64	10	SCID; DSM-IV	ACTH and cortisol, TSH	Blood, 2 days, day 1 TRH test 5 time points, day 2 DEX /CRH-test, 10 time points	IRMA	NS cortisol and ACTH,↑TSH after TRH test in recurrence	5
Zimmerman et al. [102]^c^	N	?	USA	165	47 R	40	73	0.5	Unclear; DSM-III	DEX suppression	Blood, 2 time points after DEX	RIA	NS	6
Zobel et al. [103]	N	a	Germany2	40	10 R	50	65	0.5	Unclear; DSM-IV	DEX/CRH test	Blood, 9 time points DEX/CRH-test	Unclear	↑ cortisol after DEX/CRH at discharge predicts MDD relapse	3
Zobel et al. [104]^c^	N	a	Germany2	74	13 R	50	59	0.5	Unclear; DSM-IV	DEX/CRH test, cortisol and ACTH	Blood, 5 time points DEX/CRH-test	RIA	↑ cortisol predicts MDD, ACTH NS	5
**Hormones: HPG**	**Baseline MDD excl. Y** **=** **yes, N** **=** **No**	**Baseline symptoms a** **=** **above cut-off, b** **=** **below cut-off, ?** **=** **unclear**	**Country and cohort information (nr indicates similar cohorts)**	**Total** ***N***	**Onset (O) or relapse/ recurrence (R) of MDD (N)**	**Baseline age (mean or range)**	**% female**	**Follow-up time (years; mean or max)**	**MDD diagnostic interview; Diagnostic criteria**	**Biomarker of interest**	**Measure**	**Technical details**	**Direction result of biomarker predicting onset, relapse/ recurrence of MDD**	**QA score**
Asselmann et al. [33]^a^			Germany	1760	165 O	45	50	9	DIA-X/M-CIDI	Testosterone, Androstenedione, sex hormone-binding globuline	Blood serum 8AM-7PM	Liquid-chromatography-tandem mass spectrometry and RIA	NS	6
**Hormones: HPS axis**	**Baseline MDD excl. Y** **=** **yes, N** **=** **No**	**Baseline symptoms a** **=** **above cut-off, b** **=** **below cut-off, ?** **=** **unclear**	**Country and cohort information (nr indicates similar cohorts)**	**Total** ***N***	**Onset (O) or relapse/ recurrence (R) of MDD (N)**	**Baseline age (mean or range)**	**% female**	**Follow-up time (years; mean or max)**	**MDD diagnostic interview; Diagnostic criteria**	**Biomarker of interest**	**Measurement**	**Technical details**	**Direction result of biomarker predicting onset, relapse/ recurrence of MDD**	**QA score**
Coplan et al. [47]^c^	Y	?	USA	34	13 O	15	52	9.6	K-SADS & K-SADS-E & SADS-LA; RDC	Growth hormone (sleep)	Blood over 2 nights, 72 time points	RIA	↑GH secretion	8
Franz et al. [52]^c^	N	a	USA3	29	22 R	40	100	3	SADS; RDC	Growth hormone (sleep)	Blood before onset of and during sleep, every 20 min	RIA	↑GH secretion	5
Jarrett et al. [66]^b^	N	a	USA3	29	22 R	40	100	3	SADS; RDC	Growth hormone (sleep)	Blood before onset of and during sleep, every 20 min	RIA	NS	6
Owashi et al. [84]^c^	N	b	Japan	26	6 R	57	76	0.5	Unclear; DSM-IV	Growth hormone, ACTH, cortisol	Blood after DEX/CRH and GHRH test, 5 time points	RIA	↓GH after GHRH at time of discharge. Cortisol and ACTH around DEX/CRH: NS	4
**Hormones: HPT axis**	**Baseline MDD excl. Y** **=** **yes, N** **=** **No**	**Baseline symptoms a** **=** **above cut-off, b** **=** **below cut-off, ?** **=** **unclear**	**Country and cohort information (nr indicates similar cohorts)**	**Total** ***N***	**Onset (O) or relapse/ recurrence (R) of MDD (N)**	**Baseline age (mean or range)**	**% female**	**Follow-up time (years; mean or max)**	**MDD diagnostic interview; Diagnostic criteria**	**Biomarker of interest**	**Measurement**	**Technical details**	**Direction result of biomarker predicting onset, relapse/ recurrence of MDD**	**QA score**
Joffe et al. [67]^a^	N	a	Canada	75	71 R	38	66	10	SADS-L; RDC	T4, T3, TSH	Unclear	Unclear	↓T3 was significantly related to time to recurrence. T4, TSH NS	4

Studies are sorted by the inclusion of participants with onset (O), relapse and recurrence (R) of MDD or both (O + R). The second and third columns represent information on the certainty of including healthy individuals at baseline, where the second column shows if baseline MDD diagnosis was excluded with a clinical interview at baseline, and the third column represents whether symptoms were measured with questionnaires (e.g. Hamilton depression scale, beck depression inventory) and whether participants scored above or below the questionnaires cut-off for clinical symptoms at baseline. Basic information on the demographics of participants and study measures, technical details and outcome are also given. The quality score (range 0–9) represents the overall quality of the studies, where a score > 6 represents good quality, indicative of a low risk of bias and < 4 represents poor quality, a high risk of bias.

Specific abbreviations will be mentioned per subsection

General abbreviations: *DIGS* Diagnostic Interview for Genetic Studies, *DSM* diagnostic statistical manual of mental disorders, *ICD* International Statistical Classification of Diseases and Related Health Problems, *MINI* mini-international neuropsychiatric interview, *nm* not mentioned, *NS* nonsignificant, *RDC* research diagnostic criteria, *SCAN* schedules for clinical assessment in neuropsychiatry, *SCID* Structured Clinical Interview for DSM, *K-SADS* Kiddie Schedule for Affective Disorders and Schizophrenia.

Neuroimaging abbreviations: *ACC* anterior cingulate cortex, *dmPFC* dorsal medial prefrontal cortex, *EEG* electroencephalography, *f* functional, *IFG* inferior frontal gyrus, *MFG* middle frontal gyrus, *mOFC* medial orbitofrontal cortex, *MRI* magnetic resonance imaging, orbitofrontal cortex (OFC), *PCG* precentral gyrus, *ROI* region of interest, *RSFC* resting state functional connectivity, *sg* subgenual

Gastrointestinal abbreviations: *GI* gastrointestinal, *L-5HTP* L-5-Hydroxytryptophan

Immunology abbreviations: *CAPA* Child and Adolescent Psychiatric Assessment, *CRP* c-reactive protein, *ELISA* enzyme-linked immunosorbent assay, HR hazard ratio, *IL* interleukin, *SRIF* somatostatin, *TNF* tumor necrosis factor, *YAPA* Young Adult Psychiatric Assessment, *IA* immunoassay

Neurotransmitters abbrevations: *RIA* radioimmunoassay

Neurotrophic and oxidative stress abbreviations: *ELISA* enzyme-linked immunosorbent assay, *BDNF* brain-derived neurotrophic factor

HPA abbreviations: *ACTH* adrenocorticotropic hormone, *AUC* area under the curve, *CAR* Cortisol awakening response, *CRH* Corticotropin-releasing hormone, *DEX/CRH* combined dexamethason-cortisol releasing hormone test, *DST* dexamethasone suppression test, *DHE* adehydroepiandosterone, *ELISA* enzyme-linked immunosorbent assay, *HRSD* Hamilton Rating Scale for Depression, *IA* immunoassay, *LIA* line immunoassay, *MADRS* montgomery-Asberg Depression Rating Scale, *NUFC* nocturnal urinary free cortisol, *RIA* radioimmunoassay, *TSST* trier social stress test.

HPG abbreviations: DIA-X/M-CIDI Munich-Composite International Diagnostic Interview, *RIA* radioimmunoassay

HPS: *GH* growth hormone, *GHRH* growth hormone resleasing hormone, *RIA* radioimmunoassay

HPT abbreviations: *T3* triiodothyronine, *T4* thyroxine, *TSH* thyroid stimulating hormone

^a^Not enough studies to meta-analyze

^b^Not enough data reported in this study to include in meta-analysis

^c^Included in meta-analysis

### Neuroimaging

Out of the 4210 articles screened for neuroimaging, 21 prospective biomarker studies fulfilled eligibility criteria and the update revealed 3 additional articles (total *N* = 1952, median *N* = 83, MDD development *N* = 420, median *N* = 18, range for age [6–63], % female [29–100], follow-up time [1–10], QA score (4–9)). However, due to overlap in study samples and heterogeneity in methods applied (e.g. tasks, regions of interest), meta-analysis could only be performed on some hippocampus, amygdala and frontal brain area volumes (see Table 1 and Supplementary Fig. 2). No significant odds ratios were observed for volume of the hippocampus (*N* = 3, OR = 0.660 [0.426 1.022], *p* = 0.063[54, 73, 91]), frontal brain regions (*N* = 3, OR = 0.869 [0.480 1.673], *p* = 0.730 [51, 74, 95]), nor the amygdala (*N* = 3, OR = 6.108 [0.143 261.388], *p* = 0.345 [54, 74,81]). Due to the small number of studies, no further analyses were performed.

Incidental structural MRI studies reported that both smaller and larger insula volume was significantly related to MDD development in two studies [51, 91]. No significant predictive value of the amygdala volume was found in three studies investigating two unique samples [53, 54, 74]. Two studies investigated cortical thickness in the same sample. MDD was predicted by a thinner right para-hippocampus and right fusiform gyrus but not by subcortical thickness [85, 86]. One study reported that higher ACC gray matter volume predicting MDD onset but did not report enough data for analysis [77].

Ten studies investigated if baseline brain activation predicted MDD onset, of which seven used fMRI [49, 50,76, 82, 99–101] and three used EEG [31, 39, 83]. Studies were too heterogeneous to compare. These studies showed that MDD development was predicted by: lower activity in the frontal lobe in various contexts ([39] reward task loss-gain contrast [83]; rest [71, 82]; go/nogo task, errors; [31] pre- vs posttryptophan depletion), higher activity in the insula ([99] sentence completion increasing in difficulty), higher subgenual anterior cingulate cortex (ACC) temporal and striatal connectivity ([76] self-blame vs other-blame situations) and higher mPFC activity ([50] viewing sad vs neutral movie clips). One study reported no group differences during rest [49]. Differences in subgenual ACC and MFG connectivity were also found in various regions of these networks during rest [71, 101].

### Immunology

Out of the 5603 articles screened for immunology, seven met inclusion criteria [43, 46, 61, 69, 87, 88, 94], and one additional study was identified in the update (total *N* = 27,009, median *N* = 2514, MDD development *N* = 1682, median *N* = 160, range for age (9–66), % female (43–100), follow-up time (3–12), QA score (4–9)). These studies investigated several markers for immunology: C-Reactive Protein, Interleukin-6 (IL-6), IL-1ß, Tumor Necrosis Factor-α (TNFα), Soluble Urokinase Plasminogen Activator Receptor (suPAR), 3-nitrotyrosine, and heat-shock protein 70 (HSP70) in blood or serum

CRP was the investigated in five studies with compatible measures for odds ratio [43, 46, 55, 69, 88, 94], IL (1 and or 6) in four studies, of which two studies investigated the same sample. No significant predictive effects for CRP (*N* = 4, OR = 1.557, 95% CI [0.870 2.788], *p* = 0.136) IL (*N* = 3, OR = 1.025, 95% CI [0.782 1.345], *p* = 0.856) was found. Due to the small number of studies, no further analyses were conducted.

Incidental findings were also identified. One study investigated hazard ratio and showed that CRP significantly predicted earlier time to onset or relapse/recurrence of depression [87]. In three studies (of which two investigated the same sample) TNFα was not found to predict nonsignificant were also reported [43, 55, 94]. A protein marker for inflammation SuPAR was found to predict reduced time to MDD [61]. In addition, three-nitrotyrosine and HSP70 were higher at baseline in participants that develop vs that do not develop MDD [88].

### Gastrointestinal biomarkers

Out of the 760 articles screened for the gut-related biomarkers, only one study met our inclusion criteria [40]. The study showed that children reporting symptoms of abdominal discomfort (e.g. nausea or vomiting) in response to tryptophan (L-5HTP) infusion have a higher risk of developing MDD than children who do not report these symptoms.

### Hormones

Out of the 17,114 articles screened, 38 articles were included and 1 study was identified with the update. The studies investigated the following hormonal axes: 35 hypothalamic-pituitary axis (HPA axis; the feedback loop regulation stress responses, including ACTH, CRH, CRF, cortisol), 5 hypothalamic-pituitary-gonadal-axis (HPG-axis: regulating the reproductive system e.g. DHEAS), 4 hypothalamic-pituitary-somatic axis (HPS axis: mainly regulating growth and includes growth hormone (GH)), and 3 hypothalamic-pituitary-thymus-axis (HPT axis; mainly regulating metabolism e.g. thyroid hormone). Results will be described below by these biological/hormonal axes.

### HPA axis

**Fig. 2:**
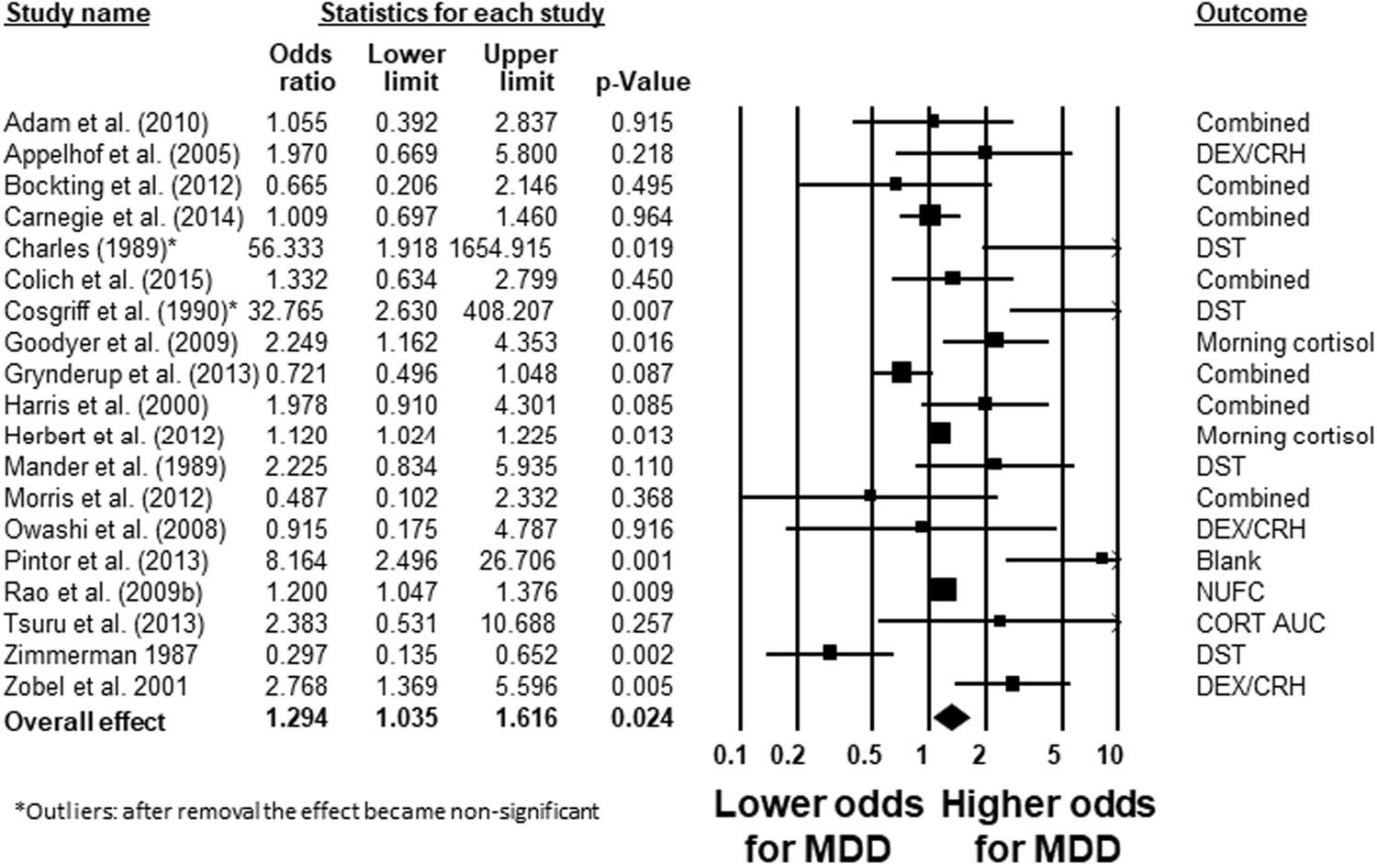
Forest plot of a meta-analysis on studies investigating measures of cortisol before MDD onset, relapse or recurrence. Charles et al. [42] and Cosgriff et al. [48] are identified as outliers, and excluding them from analysis resulted in a nonsignificant effect

The predictive value of cortisol on subsequent MDD was investigated in 35 prospective studies (total *N* = 7823, median *N* = 74, MDD development *N* = 1236, median *N* = 26, range for age (12–56), % female (44–100), follow-up time (1–22), QA score (3–9)). Cortisol was primarily measured in saliva, but differed in time of day of measurement (morning, evening, diurnal, nocturnal, reactivity), and both single time point and multiple time point measurements were included. Cortisol was a significant predictor of subsequent MDD with a small effect size (*N* = 19, OR = 1.294, 95% CI [1.035 1.616], *p* = 0.024 [30, 32, 37,41, 42, 45, 48, 58, 60, 63, 65, 78, 80, 84, 90, 92, 96, 102,104], see Fig. 2) overall comparible studies on unique samples. Heterogeneity was large and significant (76%, *p* < 0.001). The effect became nonsignificant when outliers were removed (OR = 1.228; *p* = 0.052) or low quality studies were removed (QA < 4; OR = 1.206, *p* = 0.094). Inspection of the funnel plot showed indication of publication bias (7 studies were missing on the left side), though the Eggers test was not significant *p* > 0.05. Correction for publication bias led to a nonsignificant effect. Further, the quality score of the studies moderated the effect (*β* = −0.176, *p* = 0.012) indicating a lower study quality is related to an increased effect size.

Comparing studies including participants with baseline MDD/mixed group versus no baseline MDD showed a significant higher effect size in the first group (*p* = 0.027), confirming the significance of including baseline clinical MDD diagnosis in studies (disease state effect). The pooled odds ratio for studies including baseline diagnosis was medium and significant (*N* = 13, OR = 1.919, 95% CI [1.072 1.231], *p* = 0.012), while studies excluding baseline diagnosis had a small nonsignificant pooled odds ratio (*N* = 6, OR = 1.082, 95% CI [0.938 1.249], *p* = 0.280). Comparing studies investigating onset, relapse or recurrence, or a mixed groups not significant (*p* = 0.107).

Studies investigating time until MDD onset, relapse or recurrence using Hazard ratios showed no significant predictive effect of cortisol (HR = 1.011, 95% CI [0.963 1.040], *p* = 0.447 [32, 38, 62, 79, 98]). Due to the small number of studies, no further analyses were conducted.

Besides cortisol, other HPA-axis markers in relation to relapse or recurrence of MDD were investigated incidentally. Nonsignificant findings were reported for adrenocorticotrophic hormone (ACTH) [32, 84, 89, 96, 104], and cortisol releasing hormone (CRH; [35]). One study reported lower ACTH in reaction to a DEX/CHR predicts relapse [90]. Thus, it remains unclear if HPA-axis biomarkers predict MDD development or whether results reflect disease state or quality of studies.

### HPG axis

HPG biomarkers were investigated in five studies (total *N* = 2468, median *N* = 187, MDD development *N* = 408, median *N* = 31, range for age (14–45), % female [50–100], follow-up time (1–10), QA score (6–7)). Four studies investigated dehydroepiandrosterone (DHEA) or DHEA-sulfate, (DHEAS) in saliva [56, 63, 79], but studies included the same sample and included OR and HR measures, which are not comparable. Both significant predictive effects [56, 57] as well as no significant predictive effects [63] were reported. One study showed that a higher cortisol: DHEAS ratio predicted a shorter time to recurrence [79]. One study investigated serum concentratioins of testosterone, androstenedione, and sex hormone-binding globuline (SHBG) and found no predictive effect on first onset nor the combination of onset/recurrence over 17 years [33]. Thus, it remains unclear if androsterones predict MDD development.

### HPS axis

Four studies [47, 52, 66, 84] investigated the predictive effect of GH on subsequent MDD (total *N* = 118, median *N* = 29, MDD development *n* = 23, median *N* = 22, range for age [15–57], % female [52–100], follow-up time (0.5–9.6), QA score (4–8)), of which 2 investigated the same sample and one study that did not provide sufficient data for analysis [47]. Three studies investigated GH secretion over night and a steeper increase in GH secretion was found in participants that had later onset [47] and recurrence [52] of MDD, but another study (on the same sample) found no significant predictive value for recurrence [66], and lower GH is also reported in individuals that relapse [84]. No differences were found in somatostatin (GH releasing factor) in CSF between relapsing and not relapsing participants [35]. Thus, it remains unclear if HPS markers predict MDD development.

### HPT axis

Three studies reported results investigating the HPT axis (total *N* = 113, median *N* = 25), MDD development *n* = 84, median *N* = 9, range for age [38–51], % female [54–66], follow-up time [0.25–10], QA score (4–5); [48, 67, 96]. Higher thyroid stimulating hormone (TSH) was related to recurrence in one study [96], but was also found to not differ between people with and without relapse in another study [48]. One study investigated T4, T3, and TSH using cox regression survival analyses, and reported that lower T3 was related to shorter time until relapse/recurrence [67]. Thus, the relation with HPT axis and subsequent MDD remains unclear and study quality was low.

### Oxidative stress

Out of the 1336 articles screened, 1 article met inclusion criteria [88]. Pasquali et al. [88] investigated markers for oxidative stress in blood (see Table 1). Lipid peroxidation was higher in participant that develop MDD (*N* = 37) compared to participants who did not develop MDD (*N* = 111). No significant differences between these groups were found for protein carbon and thiol content. Thus, whether oxidative stress predicts subsequent MDD remains unclear.

## Discussion

A systematic search for prospective studies investigating biomarkers of MDD onset, relapse, and recurrence was performed. Of the 67,464 articles screened, only 75 prospective studies were identified that studied biomarkers before MDD onset or relapse/recurrenc. Of those, only 38 studies reported results on participants that were healthy (had no MDD diagnosis) at baseline and are thus unconfounded by disease state. Prospective evidence for the majority of biomarkers predicting onset, and relapse/recurrence of MDD was scarce (*N* = 75) and spread over a wide range of topics: Neuroimaging (*N* = 24), Gastrointestinal factors (*N* = 1), Immunology (*N* = 8), Neurotrophic (*N* = 2), Neurotransmitters (*N* = 1), Hormones (*N* = 39), and Oxidative stress (*N* = 1). Marked heterogeneity across studies was observed for neuroimaging studies (*N* = 24). These included assessments based on EEG, task-based functional MRI, and structural MRI that focused on different brain regions, thereby precluding the calculation of an overall effect [105]. This highlights the urgent need for standardized methods in order to be able to compare data from different samples. The only significant biomarkers that increased odds for MDD onset, and relapse/recurrence was cortisol. However, the inclusion of baseline clinical diagnosis was shown to influence this effect. Therefore, the effect of disease state cannot be ruled out. Meta-analyses on CRP, TNFα, IL2&6, GH, hippocampus, amygdala, and frontal brain areas volume were not significant, potentially due to the limited amount of studies included in these analyses [range 3–4]. Only incidental (<3) studies investigated TSH, DHEAS, amygdala volumes, neurotrophic factors, oxidative stress, ACTH, neurotransmitters and gastrointestinal biomarkers. In addition, results on biomarkers were inconsistent.

Our meta-analysis showed increased cortisol had a small predictive effect on onset or relapse and recurrence of MDD, which is in line with literature showing increased cortisol levels in MDD cross-sectionally [106, 107]. Yet, this effect disappeared when studies including baseline clinical diagnoses were excluded. Since increased cortisol is also a marker of stress [108], increased cortisol may be an indirect marker of previous stressful life events or stress induced by being ill. This underlines the importance of future research following *healthy samples* without subclinical depression longitudinally until a MDD diagnosis is established. Further, cortisol results were influenced by publication bias and study quality and the effect disappeared when outliers were removed or poor quality studies were removed. This underlines the need for high-quality prospective research on biomarkes for MDD.

Some limitations of the studies included and of the meta-analyses are noted. On a study level, poor quality studies were identified and small samples that develop MDD at follow-up were investigated. Neuroimaging studies use smaller samples than immunology and hormons studies. This limits the interpretation and generalization of findings for sample size topics. Further, we did not correct for multiple testing by applying *p* = 0.05 as threshold for significance. A correction would result in disappearance of the cortisol effect, indicating this may be a false positive. Based on our narrative synthesis heterogeneity of studies was visible and studies reporting no significant results were prominent, yet tend to not report sufficient data for inclusion in meta-analysis, resulting in a bias in the meta-analyses on significant effects. These limitations may have resulted in inflated odds ratios in our meta-analysis, and results should thus be interpreted with caution.

Overall, the findings of the current systematic review highlight the lack of prospective evidence for biomarkers as predictors of onset of MDD and relapse/recurrence. Our systematic search uncovers the causality gap that is present in biomarker research. It is striking not to find strong prospective evidence for any of the postulated biological theories. Thus, most of the leading hypotheses are based on results from cross-sectional research, treatment studies, symptomatology studies, or animal studies (e.g. [8, 12, 16, 18, 20]), which cannot determine causality [21]. Whether the observed changes in putative biomarker systems in MDD is a potential cause or consequence of depression thus remains unclear.

Our results, of course, do not indicate that there are *no* causal biomarkers, but highlight the dearth of *prospective evidence* that biomarkers explain onset, and relapse/recurrence of MDD. In addition, prospective evidence would suggest causality, yet it is only the *minimum* requirement for detecting causal relations. Manipulation studies should also be performed in order to demonstrate that alteration of one variable (biomarker) leads to the expected outcome (MDD). Indeed, experimental challenges including depletion studies, such as tryptophan depletion are available and have been shown to predict depressive relapse in certain circumstances [109]. Yet, a limitation of these studies is the temporary nature of the measured outcome (e.g., brief symptom reduction) and that common higher order biological (e.g. neuromodulatory) changes may also account for the differences in depletion responses [31, 109]. Combining different techniques from different biological levels may disentangle which factors are most directly causally linked to depression etiology. Future studies applying transcranial magnetic stimulation or other brain stimulation approaches to simulate symptoms/relapse may provide more insights into causal neuroimaging biomarkers [110]. It must be noted that we did not search for relatively newly identified biomarkers, such as fatty acids [111], which are not yet part of an established etiological theory. Thus, future research is necessary to investigate if novel biomarkers can predict MDD and replicate the current incidental findings.

Notwithstanding the overall lack of prospective evidence for leading biological models for onset, relapse and recurrence of MDD, future research may be directed to focus on potential predictive biomarkers identified in a small number of studies or showing inconsistent results. These were insula volume [36], thickness [51], and activity [99, 100] frontal brain activity [31, 39, 50, 76, 82], gastrointestinal sensitivity [40], norepinephrine [68], immunology markers [61, 87], androsterones [33], and oxidative stress markers [88]. Prospective research on these biomarkers investigating development of MDD from healthy samples is needed to replicate these incidental finding and further investigate if predictive effects exist irrespectively of disease state. Indeed, there are indications that biomarkers may be causally involved, for example based on genetics research. Recent large consortium results (e.g. depression PGC [112]) have been successful in identifying genetic loci associated with depression. More importantly, depression is not a single gene disease but rather seems to be related to multiple genes in interaction with environmental factors, which lead to a spectrum of aversive outcomes, ranging from depressive symptoms to full-blown MDD [112]. The genetic loci identified explain only limited variance of depression (e.g. 2% genetic risk score [112] and mendelian randomization studies <1% [113]), whereas the heritability of MDD has been estimated at ~40% [114]. This suggests that MDD may be a more heterogeneous disorder both in etiology and pathophysiology. To unravel the biological mechanisms of MDD we therefore suggest to investiate interactions between biomarkers instead of investigating biomarkers separately for example in pathway or network approach.

In order to falsify biological theories for MDD better comparisons between or integration of studies is necessary. Open science initiatives could play a role in these efforts by enabling researchers to combine datasets over multiple cohorts (Consortia studies). However, it is noteworthy that there are large cohort samples available that allow prospective analysis on the clinical diagnosis MDD, yet clinical symptoms are more frequently investigated. In addition, baseline measurements where participants are healthy (before the development of MDD onset or relapse/recurrence) are frequently lacking in cohort studies. Further, investigating differential effects of onset versus relapse/recurrence is not common practice in biology research, whilst different mechanisms may underlie MDD onset versus maintenance. Future studies should separate samples with first onset from samples with previous episodes in order to investigate the differential mechanisms. Finally, given most theories on depression etiology include biological, psychological and social factors [115, 116], it is noteworthy that few studies have investigated combinations of these factors in a single study. Viewing depression from a more holistic perspective may help capture important interactions and improve prediction models.

## Conclusion

This systematic search for prospective evidence for biomarkers of MDD revealed scarce prospective evidence for leading biological models. Prospective evidence for etiological involvement of gastrointestinal factors, neuroimaging, neurotrophic factors, neurotransmitters, hormones (other than cortisol), immunology and oxidative stress in MDD is lacking. Cortisol was found to be a predictor for onset/relapse/recurrence of MDD, but this effect was confounded by baseline clinical depression and quality of studies. Therefore, there is a need for high quality, prospective studies on the relative contribution of biomarkers (in combination with psychosocial factors) in order to disentangle the etiology of MDD and to better understand its clinical course.

## Supplementary information

Supplemental material

## Footnotes

1 Note that this number may include duplicates since articles may be screened two times for different classes of biomarkers.
