# Supplementary material for: Prospective biomarkers of major depressive disorder: a systematic review and meta-analysis
Source: Mol Psychiatry. 2019 Nov 19;25(2):321–38. doi: 10.1038/s41380-019-0585-z (PMC6974432; doi:10.1038/s41380-019-0585-z)
Supplement: Supplementary file 1 — Supplemental material [file 41380_2019_585_MOESM1_ESM.docx]

## Supplementary material A: Search terms

## The search terms for each biological level of interest are described below. For each search the Pubmed search terms are presented. These search terms were translated into suitable terms for Embase and Psycinfo, which can be made available upon request.

## A1. Search terms: Neuroimaging

Pubmed. August 2016

("depressive disorder"[MeSH Terms] OR "depression"[MeSH Terms] OR depression[Title/Abstract] OR depressive[Title/Abstract] OR depressed[Title/Abstract] OR affective[Title/Abstract]) **AND** (recurrence[Title/Abstract] OR "recurrence"[MeSH Terms] OR recurrent[Title/Abstract] OR relapse[Title/Abstract] OR "recurrence"[MeSH Terms] OR remission[Title/Abstract] OR prognostic [Title/Abstract] OR prescriptive [Title/Abstract] OR mediator [Title/Abstract] OR moderator [Title/Abstract] OR onset [Title/Abstract] OR “first-ever” [Title/Abstract] OR predictor [Title/Abstract] OR preventi* [Title/Abstract] OR prospective [Title/Abstract] OR risk [Title/Abstract] OR vulnerability[Title/Abstract] OR longitud*[Title/Abstract] OR precipitating [Title/Abstract] OR protective [Title/Abstract] OR resilience[Title/Abstract] OR susceptibility[Title/Abstract] OR epidemiology[Title/Abstract] OR maintenance[Title/Abstract] OR "maintenance"[MeSH Terms] OR resistance[Title/Abstract] OR chronicity[Title/Abstract] OR Persistence[Title/Abstract] OR "Chronic depression"[Title/Abstract] OR "treatment resistant depression"[Title/Abstract]) **AND** ("fMRI"[Title/Abstract] OR "functional MRI"[Title/Abstract] OR "functional magnetic"[Title/Abstract] OR MRI[Title/Abstract] OR "magnetic resonance imaging"[Title/Abstract] OR EEG[Title/Abstract] OR "electroencephalography"[Title/Abstract] OR "electroencephalogram"[Title/Abstract] OR DTI[Title/Abstract] OR "diffusion tensor imaging"[Title/Abstract] OR  "brain imaging"[Title/Abstract] OR MEG[Title/Abstract] OR "magnetoencephalography"[Title/Abstract] OR "magnetoencephalogram"[Title/Abstract] OR neuroimaging[mesh] OR PET[Title/Abstract] OR "Positron emission tomography"[Title/Abstract] OR SPECT[Title/Abstract] OR "Single-photon emission computed tomography"[Title/Abstract] OR "Computed tomography"[Title/Abstract] OR "Computed Axial Tomography"[Title/Abstract]) **AND** Humans[Mesh] **AND** English[lang] **NOT** ("review"[Publication Type] OR "review literature as topic"[MeSH Terms] OR "review"[All Fields])

**A2. Search terms: Gastro-intestinal factors**

Pubmed. February 2017

(microbiota[MeSH Terms] OR microbiome [MESH] OR fecal:ab.ti OR probiotica:ab.ti OR prebiotic:ab.ti OR "gut-brain":ab.ti OR "gut microbiome":ab.ti OR "gut microbiota"[tiab] OR gut[tiab]) **AND** ("depressive disorder"[MeSH Terms] OR "depression"[MeSH Terms] OR depression[Title/Abstract] OR depressive[Title/Abstract] OR depressed[Title/Abstract] OR affective[Title/Abstract]) **AND** (recurrence[Title/Abstract] OR "recurrence"[MeSH Terms] OR recurrent[Title/Abstract] OR relapse[Title/Abstract] OR "recurrence"[MeSH Terms] OR remission[Title/Abstract] OR prognostic[Title/Abstract] OR prescriptive[Title/Abstract] OR mediator[Title/Abstract] OR moderator[Title/Abstract] OR onset[Title/Abstract] OR "first-ever"[Title/Abstract] OR predictor[Title/Abstract] OR prospective[Title/Abstract] OR risk[Title/Abstract] OR vulnerability[Title/Abstract] OR longitudinal[Title/Abstract] OR precipitating[Title/Abstract] OR protective[Title/Abstract] OR resilience[Title/Abstract] OR susceptibility[Title/Abstract] OR epidemiology[Title/Abstract]) **AND** English[lang] **NOT** ("review"[Publication Type] OR "review"[All Fields])

**A3 Search terms: Immunology**

Pubmed. Februari 2017

(CRP[tiab] OR "C-reactive protein"[tiab] OR "C-reactive protein"[MeSH Terms] OR ESR[tiab] OR erythrocyte[tiab] OR albumin[tiab] OR "interferon-a"[tiab] OR thrombocyte[tiab] OR (cytokine[tiab] OR cytokine'[tiab] OR cytokine's[tiab] OR ("cytokines"[MeSH Terms] OR "cytokines"[All Fields])) OR "Tumor Necrosis Factor-alpha"[Mesh] OR "Interleukin-1"[Mesh] OR "Interleukin-4"[Mesh] OR "Interleukin-6"[Mesh] OR "Interleukin-8"[Mesh] OR "Interleukin-10"[Mesh] OR "Receptors. Interleukin-2"[Mesh] OR "Chemokine CCL2"[Mesh] OR "Chemokine CCL3"[Mesh] OR "Chemokine CCL11"[Mesh] OR CXCL-8[Title/Abstract] OR CXCL-10[Title/Abstract]) **AND** ("depressive disorder"[MeSH Terms] OR "depression"[MeSH Terms] OR depression[Title/Abstract] OR depressive[Title/Abstract] OR depressed[Title/Abstract] OR affective[Title/Abstract]) **AND** "humans"[MeSH Terms] **AND** (recurrence[Title/Abstract] OR "recurrence"[MeSH Terms] OR recurrent[Title/Abstract] OR relapse[Title/Abstract] OR "recurrence"[MeSH Terms] OR remission[Title/Abstract] OR prognostic[Title/Abstract] OR prescriptive[Title/Abstract] OR mediator[Title/Abstract] OR moderator[Title/Abstract] OR onset[Title/Abstract] OR "first-ever"[Title/Abstract] OR predictor[Title/Abstract] OR prospective[Title/Abstract] OR risk[Title/Abstract] OR vulnerability[Title/Abstract] OR longitudinal[Title/Abstract] OR precipitating[Title/Abstract] OR protective[Title/Abstract] OR resilience[Title/Abstract] OR susceptibility[Title/Abstract] OR epidemiology[Title/Abstract]) **AND** "humans"[MeSH Terms] **AND** English[lang] **NOT** ("review"[Publication Type] OR "review literature as topic"[MeSH Terms] OR "review"[All Fields])

## A4 Search terms: Neurotrophic factors

Pubmed. July 2017

("depressive disorder"[MeSH Terms]” OR “"depression"[MeSH Terms]” OR “depression[Title/Abstract]” OR “depressive[Title/Abstract]” OR “depressed[Title/Abstract]” OR “affective[Title/Abstract]) AND ((recurrence[Title/Abstract]” OR “"recurrence"[MeSH Terms]” OR “recurrent[Title/Abstract]” OR “relapse[Title/Abstract]” OR “"recurrence"[MeSH Terms]” OR “remission[Title/Abstract]” OR “prognostic [Title/Abstract]” OR “prescriptive [Title/Abstract]” OR “mediator [Title/Abstract]” OR “moderator [Title/Abstract]” OR “onset [Title/Abstract]” OR ““first-ever” [Title/Abstract]” OR “predictor [Title/Abstract]” OR “preventi* [Title/Abstract]” OR “prospective [Title/Abstract]” OR “risk [Title/Abstract]” OR “vulnerability[Title/Abstract]” OR “longitud*[Title/Abstract]” OR “precipitating [Title/Abstract]” OR “protective [Title/Abstract]” OR “resilience[Title/Abstract]” OR “susceptibility[Title/Abstract]” OR “epidemiology[Title/Abstract]” OR “maintenance[Title/Abstract]” OR “"maintenance"[MeSH Terms]” OR “resistance[Title/Abstract]” OR “chronicity[Title/Abstract]” OR “Persistence[Title/Abstract]” OR “"Chronic depression"[Title/Abstract]” OR “"treatment resistant depression"[Title/Abstract])) (BDNF[mesh] OR neurotroph*[tiab] OR "growth factor"[tiab] OR "growth protein"[tiab]

## A5 Search terms: Neurotransmitters

## Pubmed. July 2016

"depressive disorder"[MeSH Terms] OR "depression"[MeSH Terms] OR depression[tiab] OR depressive[tiab] OR depressed[tiab] OR affective[tiab] **AND** "serotonin"[MeSH Terms] OR “serotonin”[tiab] ("2014"[Date - Publication] : "2016/08/02"[Date - Publication]) OR "dopamine"[MeSH Terms] OR “dopamine”[tiab] ("2014"[Date - Publication] : "2016/08/02"[Date - Publication]) OR norepinephrine[mesh] OR noradrenalin [mesh] OR (norepinephrine[tiab] OR noradrenalin[tiab])("2014"[Date - Publication] : "2016/08/02"[Date - Publication]) OR "gamma-Aminobutyric acid"[MeSH Terms]) OR (“gamma-Aminobutyric acid”[tiab] ("2014"[Date - Publication] : "2016/08/02"[Date - Publication]) OR "glutamic acid"[MeSH Terms] OR "glutamates"[MeSH Terms]) OR “glutamic acid”[tiab] OR “glutamates”[tiab])("2014"[Date - Publication] : "2016/08/02"[Date - Publication]) OR “acetylcholine"[MeSH Terms] OR acetylcholin* OR “cholinergic”[MeSH Terms] OR choliner* ) **AND** "humans"[MeSH Terms]

Comment: both mesh terms and title abstract searches are performed. To reduce the number of articles to be screened, we searched for title/abstract only over the most recent years. since these articles may not be indexed by mesh terms. This criterion was not applied for the other search engines.

## A6 Search terms: Hormones

Initial search on gonadal hormones. Pubmed. November 2016

(("depressive disorder"[MeSH Terms] OR "depression"[MeSH Terms] OR depression[Title/Abstract] OR depressive[Title/Abstract] OR depressed[Title/Abstract] OR affective[Title/Abstract]) **AND** (OR "sex hormones"[Title/Abstract] OR “sex steroids”[Title/Abstract] OR "gonadal steroid hormones"[MeSH Terms] OR "gonadal steroid hormones"[Title/Abstract] OR “gonadal steroids”[Title/Abstract] OR estradiol[MeSH Terms] OR estrad*[Title/Abstract] OR estradiol[Title/Abstract] OR estrone[MeSH Terms] OR estron*[Title/Abstract] OR estrone[Title/Abstract] OR oestrogen[MeSH Terms] OR estrogen[MeSH Terms] OR oestrogen[Title/Abstract] OR estrogen[Title/Abstract] OR oestradiol[MeSH Terms]OR oestradiol[Title/Abstract] OR oestrad*[Title/Abstract] OR progesterone[MeSH Terms] OR progesteron[Title/Abstract] OR progest*[Title/Abstract] OR prolactin[MeSH Terms] OR prolactin[Title/Abstract] OR prolact*[Title/Abstract] OR androgens[MeSH Terms] OR androgens[Title/Abstract] OR androgen*[Title/Abstract] OR testosterone[MeSH Terms] OR testost*[Title/Abstract] OR dehydroepiandrosterone[Title/Abstract] OR dehydroepiandrosterone[MeSH Terms] OR DHEA[MeSH Terms] OR DHEA[Title/Abstract] OR gonadotropins[Title/Abstract] OR gonadotrop*[Title/Abstract] OR “follicle stimulating hormone” [MeSH Terms] OR “follicle stimulating hormone” [Title/Abstract] OR FSH[Title/Abstract] OR “luteinizing hormone”[MeSH Terms] OR “luteinizing hormone”[Title/Abstract]

Pubmed additional search. other hormones. May 2017

(("depressive disorder"[MeSH Terms] OR "depression"[MeSH Terms] OR depression[Title/Abstract] OR depressive[Title/Abstract] OR depressed[Title/Abstract] OR affective[Title/Abstract]) **AND** ((recurrence[Title/Abstract] OR "recurrence"[MeSH Terms] OR recurrent[Title/Abstract] OR relapse[Title/Abstract] OR "recurrence"[MeSH Terms] OR remission[Title/Abstract] OR prognostic [Title/Abstract] OR prescriptive [Title/Abstract] OR mediator [Title/Abstract] OR moderator [Title/Abstract] OR onset [Title/Abstract] OR “first-ever” [Title/Abstract] OR predictor [Title/Abstract] OR preventi* [Title/Abstract] OR prospective [Title/Abstract] OR risk [Title/Abstract] OR vulnerability[Title/Abstract] OR longitud*[Title/Abstract] OR precipitating [Title/Abstract] OR protective [Title/Abstract] OR resilience[Title/Abstract] OR susceptibility[Title/Abstract] OR epidemiology[Title/Abstract] OR maintenance[Title/Abstract] OR "maintenance"[MeSH Terms] OR resistance[Title/Abstract] OR chronicity[Title/Abstract] OR Persistence[Title/Abstract] OR "Chronic depression"[Title/Abstract] OR "treatment resistant depression"[Title/Abstract])) **AND** Cortisol[tiab] or cortisol[mesh] OR "Corpus Luteum Hormones" OR "Epidermal Growth Factor" OR "Gastric Inhibitory Polypeptide" OR "Gastrin-Releasing Peptide" OR "insuline-like growth factor" OR "Natriuretic Peptides" OR "Parathyroid Hormone-Related Protein" OR "Parathyroid Hormone" OR "Thymic Factor" OR "Vasoactive Intestinal Peptide" OR 17-Ketosteroids OR ACTH OR Activins OR ADH OR Adipokines OR adrenalin OR Adrenomedullin OR angiotensin OR Angiotensins OR ANP OR Bombesin OR Calcitonin OR Cholecystokinin OR corticotropin OR Dextrothyroxine OR Diiodotyrosine OR dimethyltryptamine OR endorphin OR epo OR erytropoetin OR gastrin OR Gastrins OR ghrelin OR glucagon OR Glucocorticoids OR Gonadotropins OR growth hormone OR histamine OR Hydroxycorticosteroids OR IGF1 OR Inhibins OR Inhibins OR insulin OR leptin OR LH OR Melatonin OR Monoiodotyrosine OR Motilin OR oxytocin OR Peptide PHI OR Peptide YY OR Proglucagon OR Relaxin OR renin OR Secretin OR somatostatin OR T3 OR T4 OR triiodothyronine OR Thymopoietins OR Thymosin OR Thyroid OR Thyronines OR Thyroxine OR TRH OR TSH OR Urocortin OR Urotensins OR vasopressin OR sterols OR cholesterol OR "Corpus Luteum Hormones"[Title/Abstract] OR "Epidermal Growth Factor"[Title/Abstract] OR "Gastric Inhibitory Polypeptide"[Title/Abstract] OR "Gastrin-Releasing Peptide"[Title/Abstract] OR "insuline-like growth factor"[Title/Abstract] OR "Natriuretic Peptides"[Title/Abstract] OR "Parathyroid Hormone-Related Protein"[Title/Abstract] OR "Parathyroid Hormone"[Title/Abstract] OR "Thymic Factor"[Title/Abstract] OR "Vasoactive Intestinal Peptide"[Title/Abstract] OR 17-Ketosteroids[Title/Abstract] OR ACTH [Title/Abstract] OR Activins[Title/Abstract] OR ADH [Title/Abstract] OR Adipokines[Title/Abstract] OR adrenalin[Title/Abstract] OR Adrenomedullin[Title/Abstract] OR angiotensin[Title/Abstract] OR Angiotensins[Title/Abstract] OR ANP [Title/Abstract] OR Bombesin[Title/Abstract] OR Calcitonin[Title/Abstract] OR Cholecystokinin[Title/Abstract] OR corticotropin[Title/Abstract] OR Dextrothyroxine[Title/Abstract] OR Diiodotyrosine[Title/Abstract] OR dimethyltryptamine[Title/Abstract] OR endorphin[Title/Abstract] OR epo[Title/Abstract] OR erytropoetin[Title/Abstract] OR gastrin[Title/Abstract] OR Gastrins[Title/Abstract] OR ghrelin[Title/Abstract] OR glucagon[Title/Abstract] OR Glucocorticoids[Title/Abstract] OR Gonadotropins[Title/Abstract] OR growth hormone[Title/Abstract] OR histamine[Title/Abstract] OR Hydroxycorticosteroids[Title/Abstract] OR IGF1[Title/Abstract] OR Inhibins[Title/Abstract] OR Inhibins[Title/Abstract] OR insulin[Title/Abstract] OR leptin[Title/Abstract] OR LH[Title/Abstract] OR Melatonin[Title/Abstract] OR Monoiodotyrosine[Title/Abstract] OR Motilin[Title/Abstract] OR oxytocin[Title/Abstract] OR Peptide PHI[Title/Abstract] OR Peptide YY[Title/Abstract] OR Proglucagon[Title/Abstract] OR Relaxin[Title/Abstract] OR renin[Title/Abstract] OR Secretin[Title/Abstract] OR somatostatin[Title/Abstract] OR T3[Title/Abstract] OR T4[Title/Abstract] OR triiodothyronine[Title/Abstract] OR Thymopoietins[Title/Abstract] OR Thymosin[Title/Abstract] OR Thyroid[Title/Abstract] OR Thyronines[Title/Abstract] OR Thyroxine[Title/Abstract] OR TRH[Title/Abstract] OR TSH[Title/Abstract] OR Urocortin[Title/Abstract] OR Urotensins[Title/Abstract] OR vasopressin[Title/Abstract] OR sterols[Title/Abstract] OR cholesterol[Title/Abstract] **AND** (English[lang] **NOT** ("review"[Publication Type] OR "review literature as topic"[MeSH Terms])) **AND** human[mesh]

**A7 Oxidative stress**

Pubmed search. July 2017

("depressive disorder"[MeSH Terms]” OR “"depression"[MeSH Terms]” OR “depression[Title/Abstract]” OR “depressive[Title/Abstract]” OR “depressed[Title/Abstract]” OR “affective[Title/Abstract]) **AND** ((recurrence[Title/Abstract]” OR “"recurrence"[MeSH Terms]” OR “recurrent[Title/Abstract]” OR “relapse[Title/Abstract]” OR “"recurrence"[MeSH Terms]” OR “remission[Title/Abstract]” OR “prognostic [Title/Abstract]” OR “prescriptive [Title/Abstract]” OR “mediator [Title/Abstract]” OR “moderator [Title/Abstract]” OR “onset [Title/Abstract]” OR ““first-ever” [Title/Abstract]” OR “predictor [Title/Abstract]” OR “preventi* [Title/Abstract]” OR “prospective [Title/Abstract]” OR “risk [Title/Abstract]” OR “vulnerability[Title/Abstract]” OR “longitud*[Title/Abstract]” OR “precipitating [Title/Abstract]” OR “protective [Title/Abstract]” OR “resilience[Title/Abstract]” OR “susceptibility[Title/Abstract]” OR “epidemiology[Title/Abstract]” OR “maintenance[Title/Abstract]” OR “"maintenance"[MeSH Terms]” OR “resistance[Title/Abstract]” OR “chronicity[Title/Abstract]” OR “Persistence[Title/Abstract]” OR “"Chronic depression"[Title/Abstract]” OR “"treatment resistant depression"[Title/Abstract])) **AND** (oxidative stress [Mesh] OR reactive oxygen species OR free radicals OR nitric oxideOR lipid peroxidation OR malondialdehyde OR thiobarbituric acid reactive substances OR protein carbonyl OR protein carbonylation OR glutathione OR [deoxyguanosine](http://www.ncbi.nlm.nih.gov/mesh/68003849) [MeSH] OR 8-hydroxy-2'-deoxyguanosine [Supplementary Concept] OR 8-oxo-7OR8-dihydrodeoxyguanine [Supplementary Concept] OR deoxyguanosine OR 8-hydroxy-2-deoxyguanosine OR 8'-hydroxy-2'-deoxyguanosine OR 8-hydroxy-2'-deoxyguanosine OR 8-hydroxydeoxyguanosine OR 8-hydroxy-deoxyguanosine OR 8OHdG OR 8-OHdG OR 8-OH-dG OR 2'-deoxy-8-hydroxyguanosine OR 8-oxo-2-deoxyguanosine OR oxo8dG OR 8-oxodG OR 8-oxo-dG OR 8-oxodGuo OR 7OR8-dihydro-8-oxo-2'-deoxyguanosine OR 8-oxo-7OR8-dihydrodeoxyguanine OR 8-oxo-7OR8-dihydro-2'-deoxyguanosine OR 8-OHGua OR 8-oxoGua OR 8-Oxo-guanine OR 8-oxoG OR 8-hydroxyguanine OR 8-OHG OR 8-hydroxy-2’-guanosine OR 8-hydroxyguanosine OR Isoprostanes [MESH] OR F2 isoprostanes [MESH] OR [Dinoprost](http://www.ncbi.nlm.nih.gov/mesh/68015237)/analogs and derivatives [MESH] OR 8-epi-prostaglandin F2alpha [Supplementary Concept] OR Isoprostane OR Isoprostanes OR F2 isoprostane OR F2 isoprostanes OR F2 IsoP OR F2 IsoPs OR 8-isoprostane OR 8-iso-PGF OR 8-iso-PGF2a OR 8-iso-PGF 2a OR 8-iso-PGF2alpha OR 8-iso-PGF 2alpha OR 8-iso-prostaglandin F2alpha OR 8-isoprostaglandin F2alpha OR 8-epiprostaglandin F2alpha OR 8-epi-PGF2alpha OR 8-epi-PGF2 alpha OR PGF2 OR PGF2alpha OR Prostaglandin F2 OR Prostaglandin F2alpha OR Prostaglandin F2 alpha OR F2alpha Prostaglandin OR F2 alpha Prostaglandin)

## Supplementary material B: Flow-charts per search

## *Supplementary Table 1:* Horizontal flowchart for each biological levels of interest and the search over all biomarkers to update the meta to June 2019.

|  | **Records identified** | **Additional records** | **Duplicates removed** | **Records ti/ab screened** | **Records excluded** | **Full text screened** | **Exclusion with reasons** | **Exclusion total** | **Prospective studies** | **Inclusions** |
| --- | --- | --- | --- | --- | --- | --- | --- | --- | --- | --- |
| **Neuroimaging** | 7345 | 14 | 4222 | 4222 | 4184 | 40 | 10 no MDD; 8 not prospective; 1 not original | 19 | 21 | 15 |
| **Gastro-intestinal** | 699 | 1 | 700 | 700 | 695 | 5 | 2 no MDD; 1 no biomarker; 1 not prospective | 4 | 1 | 1 |
| **Immunology** | 7335 | 6 | 5374 | 5374 | 5363 | 13 | 2 no MDD; 1 no biomarker; 2 not prospective; 1 indirect analysis | 6 | 7 | 3 |
| **Neurotrophic** | 1530 | 1 | 1380 | 1380 | 1369 | 12 | 3 no MDD; 1 no biomarker; 5 not prospective for onset relapse and recurrence; 1 mixed group | 10 | 2 | 1 |
| **Neurotransmitters** | 20430 | 0 | 16960 | 16960 | 16553 | 417 | 30 no MDD; 119 no biomarker; 237 not prospective; 19 not original; 4 no full text; 7 language | 416 | 1 | 0 |
| **Hormones** | 23597 | 7 | 17114 | 17114 | 16953 | 141 | 57 no MDD; 3 no biomarker; 37 not prospective; 1 mixed group; 5 no full text | 103 | 38 | 14 |
| **Oxidative stress** | 1581 | 1 | 1310 | 1336 | 1331 | 5 | 1 not prospective; 3 no oxidative stress | 4 | 1 | 0 |
| **Update August 2016 – June 2019 over all topics combined** | 4945 | 3 | 4757 | 4757 | 9624 | 78 | 20 no MDD; 14 no biomarker; 33 not prospective; 1 not original | 67 | 9 | Neuroimaging: 7; Immunology: 1; Hormones: 1. |

ti/ab= title/abstract.

## Supplementry material C: PRISMA checklist(1)

| **Section/topic** | **#** | **Checklist item** | **Reported on page #** |
| --- | --- | --- | --- |
| **TITLE** | | |  |
| Title | 1 | Identify the report as a systematic review, meta-analysis, or both. | 1 |
| **ABSTRACT** | | |  |
| Structured summary | 2 | Provide a structured summary including, as applicable: background; objectives; data sources; study eligibility criteria, participants, and interventions; study appraisal and synthesis methods; results; limitations; conclusions and implications of key findings; systematic review registration number. | 2 |
| **INTRODUCTION** | | |  |
| Rationale | 3 | Describe the rationale for the review in the context of what is already known. | 3-4 |
| Objectives | 4 | Provide an explicit statement of questions being addressed with reference to participants, interventions, comparisons, outcomes, and study design (PICOS). | 3-4 |
| **METHODS** | | |  |
| Protocol and registration | 5 | Indicate if a review protocol exists, if and where it can be accessed (e.g., Web address), and, if available, provide registration information including registration number. | 4 |
| Eligibility criteria | 6 | Specify study characteristics (e.g., PICOS, length of follow-up) and report characteristics (e.g., years considered, language, publication status) used as criteria for eligibility, giving rationale. | 4-5 |
| Information sources | 7 | Describe all information sources (e.g., databases with dates of coverage, contact with study authors to identify additional studies) in the search and date last searched. | 4-5 |
| Search | 8 | Present full electronic search strategy for at least one database, including any limits used, such that it could be repeated. | Supplementary material |
| Study selection | 9 | State the process for selecting studies (i.e., screening, eligibility, included in systematic review, and, if applicable, included in the meta-analysis). | 4-6 |
| Data collection process | 10 | Describe method of data extraction from reports (e.g., piloted forms, independently, in duplicate) and any processes for obtaining and confirming data from investigators. | 5 |
| Data items | 11 | List and define all variables for which data were sought (e.g., PICOS, funding sources) and any assumptions and simplifications made. | 5 |
| Risk of bias in individual studies | 12 | Describe methods used for assessing risk of bias of individual studies (including specification of whether this was done at the study or outcome level), and how this information is to be used in any data synthesis. | 5-6 |
| Summary measures | 13 | State the principal summary measures (e.g., risk ratio, difference in means). | 5-6 |
| Synthesis of results | 14 | Describe the methods of handling data and combining results of studies, if done, including measures of consistency (e.g., I^2^) for each meta-analysis. | 5-6 |

Page 1 of 2

| **Section/topic** | **#** | **Checklist item** | **Reported on page #** |
| --- | --- | --- | --- |
| Risk of bias across studies | 15 | Specify any assessment of risk of bias that may affect the cumulative evidence (e.g., publication bias, selective reporting within studies). | 5-6 |
| Additional analyses | 16 | Describe methods of additional analyses (e.g., sensitivity or subgroup analyses, meta-regression), if done, indicating which were pre-specified. | 5-6 |
| **RESULTS** | | |  |
| Study selection | 17 | Give numbers of studies screened, assessed for eligibility, and included in the review, with reasons for exclusions at each stage, ideally with a flow diagram. | 6 and Figure 1 |
| Study characteristics | 18 | For each study, present characteristics for which data were extracted (e.g., study size, PICOS, follow-up period) and provide the citations. | 6-11, and Table 1 |
| Risk of bias within studies | 19 | Present data on risk of bias of each study and, if available, any outcome level assessment (see item 12). | 6-11, Table 1 and Supplementary table 1 |
| Results of individual studies | 20 | For all outcomes considered (benefits or harms), present, for each study: (a) simple summary data for each intervention group (b) effect estimates and confidence intervals, ideally with a forest plot. | 7-11, Figure 2 and supplementary Figure 2 |
| Synthesis of results | 21 | Present results of each meta-analysis done, including confidence intervals and measures of consistency. | 7-11, Supplementary figure 2, Supplementary table 1 |
| Risk of bias across studies | 22 | Present results of any assessment of risk of bias across studies (see Item 15). | Table 1 Supplementary table 1 |
| Additional analysis | 23 | Give results of additional analyses, if done (e.g., sensitivity or subgroup analyses, meta-regression [see Item 16]). | 9-10 and Supplementary table 2 |
| **DISCUSSION** | | |  |
| Summary of evidence | 24 | Summarize the main findings including the strength of evidence for each main outcome; consider their relevance to key groups (e.g., healthcare providers, users, and policy makers). | 11-12 |
| Limitations | 25 | Discuss limitations at study and outcome level (e.g., risk of bias), and at review-level (e.g., incomplete retrieval of identified research, reporting bias). | 12 |
| Conclusions | 26 | Provide a general interpretation of the results in the context of other evidence, and implications for future research. | 10-12 |
| **FUNDING** | | |  |
| Funding | 27 | Describe sources of funding for the systematic review and other support (e.g., supply of data); role of funders for the systematic review. | 1 |

*Supplementary Figure 1:* Graphic overview of biological areas of interest for this systematic overview of prospective biomarkers for depression.

*
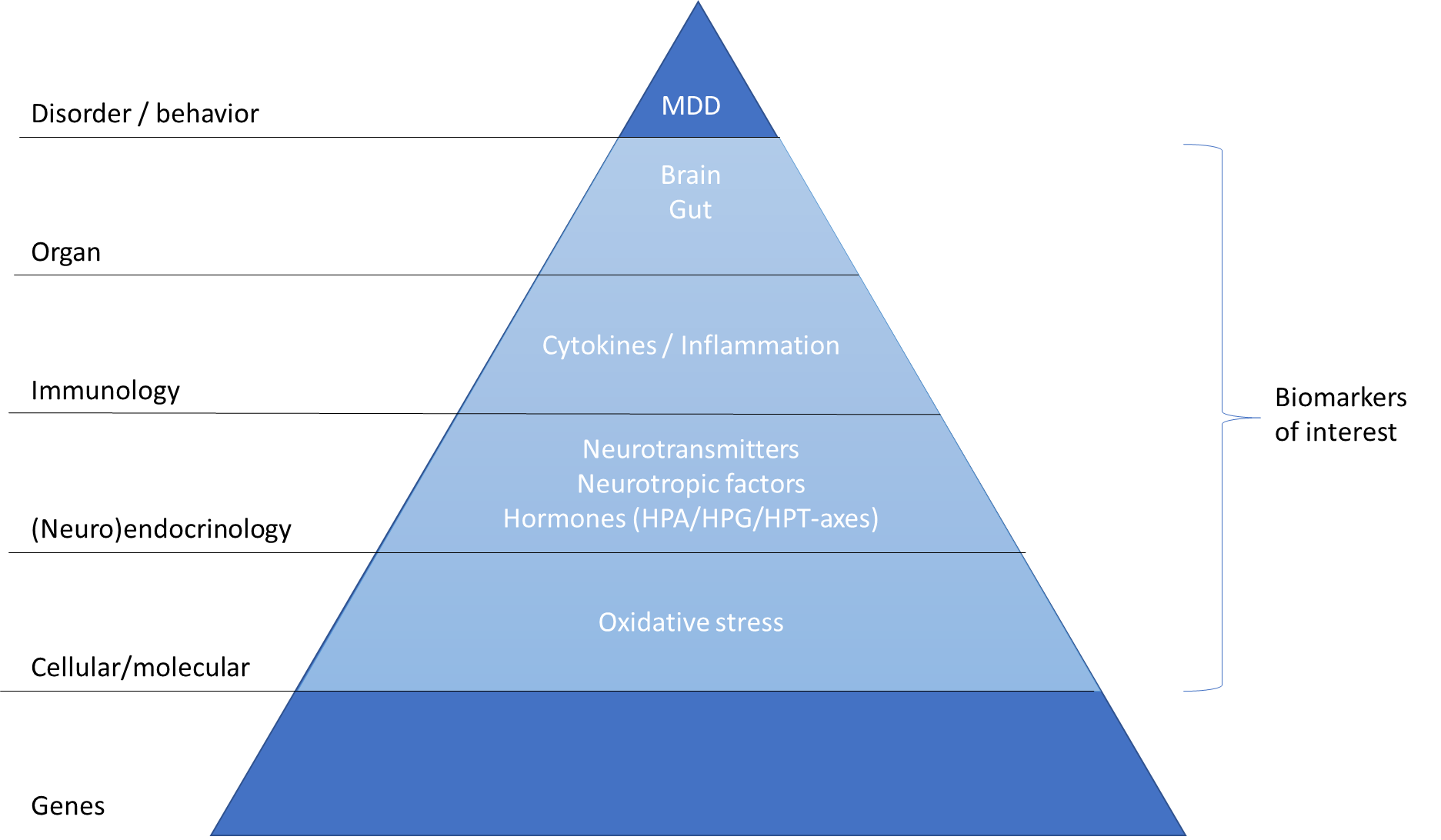
Supplementary Figure 2*

Forest plots of meta-analyses for neuroimaging, immunology, and hormones. The top forest plots represent odds ratios, the bottom plot represents hazard ratios.

**

*Supplementary Table 1:* Quality assessment of included studies (n=70) sorted alphabetically by first author.

|  |  | 1. Was selection of exposed (MDD development) and nonexposed (no MDD) cohorts drawn from the same population? | 2. Can we be confident in the assessment of MDD? | 3. Can we be confident that the outcome of interest (MDD diagnosis) was not present at start of study? | 4. Did the study match exposed and unexposed for all variables that are associated with the outcome of interest or did the statistical analysis adjust for these prognostic variables | 5. Can we be confident in the assessment of the presence or absence of prognostic factors (Biomarkers of interest)? | 6. Can we be confident in the assessment of outcome (MDD diagnosis)? | 7. Was the follow up of cohorts adequate? | 8. Were co-interventions similar between groups? | 9. Other sources of bias: Was the study apparently free of other problems that could put it at risk of bias? | Quality score (Total number +) |
| --- | --- | --- | --- | --- | --- | --- | --- | --- | --- | --- | --- |
| 1 | Adam et al. (2010) | + | + | - | + | + | + | + | ? | + | 7 |
| 2 | Allen et al. (2009) | + | + | + | + | + | ? | ? | ? | + | 6 |
| 3 | Appelhof et al. (2006) | + | + | - | + | + | + | + | ? | - | 6 |
| 4 | Asselmann et al. (2019) | + | ? | + | ? | + | + | + | ? | + | 6 |
| 5 | Aubry et al. (2007) | + | + | + | + | + | + | + | ? | + | 8 |
| 6 | Banki et al. (1992) | + | ? | - | ? | + | ? | + | ? | - | 3 |
| 7 | Belden et al. (2015) | + | + | + | + | + | + | ? | ? | - | 6 |
| 8 | Bockting et al. (2012) | + | + | + | + | + | + | ? | ? | + | 7 |
| 9 | Bouhuys et al. (2006) | + | + | - | + | + | ? | ? | + | + | 6 |
| 10 | Bress et al. (2013) | + | + | + | + | + | ? | + | ? | + | 7 |
| 11 | Campo et al. 2003 | + | + | + | ? | + | + | ? | ? | + | 6 |
| 12 | Carnegie et al. (2014) | + | ? | - | + | + | ? | + | ? | + | 5 |
| 13 | Charles et al. (1989) | + | + | - | + | ? | ? | + | ? | - | 4 |
| 14 | Chocano-Bedoya et al. (2014) | + | - | - | + | ? | - | + | ? | + | 4 |
| 15 | Chopra et al. (2008) | + | + | + | + | ? | - | ? | ? | - | 4 |
| 16 | Colich et al. (2015) | + | + | + | + | + | + | + | + | + | 9 |
| 17 | Copeland et al. (2012) | + | + | + | + | + | + | + | + | + | 9 |
| 18 | Coplan et al. 2000 | + | + | + | + | + | + | + | ? | + | 8 |
| 19 | Cosgriff et al. (1990) | + | - | - | + | + | ? | ? | + | + | 5 |
| 20 | Davey et al. (2015) | + | + | + | ? | + | ? | - | ? | - | 4 |
| 21 | Farb et al. (2011) | ? | + | + | ? | + | + | + | + | + | 7 |
| 22 | Foland-Ross et al. (2015) | + | + | + | + | + | ? | + | ? | + | 7 |
| 23 | Franz et al. (1999) | - | + | - | + | + | ? | - | + | + | 5 |
| 24 | Frodl et al. (2004) | ? | - | - | + | + | + | + | + | + | 6 |
| 25 | Frodl et al. (2008) | ? | - | - | + | + | + | + | + | + | 6 |
| 26 | Glaus et al. (2018) | + | + | ? | + | + | + | + | + | - | 7 |
| 27 | Goodyer et al. (2000a) | + | + | ? | + | + | ? | + | ? | + | 6 |
| 28 | Goodyer et al. (2000b) | + | + | ? | + | + | ? | + | + | + | 7 |
| 29 | Goodyer et al. (2009) | + | + | ? | + | + | ? | + | ? | + | 6 |
| 30 | Goodyer et al. (2010) | + | + | ? | + | + | ? | + | ? | + | 6 |
| 31 | Grynderup et al. (2013) | + | + | + | + | + | + | + | + | + | 9 |
| 32 | Haastrup et al. (2014) | + | - | - | + | + | ? | + | + | + | 6 |
| 33 | Hardeveld et al. (2014) | + | + | + | + | + | ? | ? | + | + | 7 |
| 34 | Harris et al. (2000) | + | + | ? | + | + | ? | + | ? | + | 6 |
| 35 | Hatzinger et al. (2002) | + | + | - | + | + | ? | + | + | + | 7 |
| 36 | Herbert et al. (2012) | + | + | + | + | + | ? | + | + | + | 8 |
| 37 | Jarrett et al. (1994) | - | + | - | + | + | ? | + | + | + | 6 |
| 38 | Joffe et al. (2000) | + | + | - | ? | ? | + | + | ? | - | 4 |
| 39 | Johnston et al. (1999) | + | + | - | - | + | + | + | ? | + | 6 |
| 40 | Khandaker et al. (2014) | + | ? | - | + | + | - | + | ? | + | 5 |
| 41 | Kronmuller et al. (2008) | ? | + | - | + | + | ? | + | ? | + | 5 |
| 42 | Langenecker et al. (2018) | + | + | + | + | + | + | + | + | - | 8 |
| 43 | LeMoult et al. (2015) | + | + | + | + | + | + | + | + | + | 9 |
| 44 | Little et al. (2014) | + | + | + | + | + | ? | ? | ? | + | 6 |
| 45 | Little et al. (2015) | + | + | ? | + | + | ? | + | ? | - | 5 |
| 46 | Lok et al. (2012) | + | + | + | + | - | + | + | + | + | 8 |
| 47 | Lythe et al. (2015) | + | + | + | + | + | + | + | + | + | 9 |
| 48 | Macoveanu et al. (2018) | + | + | + | ? | + | - | + | ? | - | 5 |
| 49 | Mander et al. (1989) | + | + | - | + | + | ? | + | - | - | 5 |
| 50 | Mocking et al. (2013) | + | + | + | ? | + | ? | + | ? | + | 6 |
| 51 | Morris et al. (2012) | + | + | + | + | + | + | + | ? | + | 8 |
| 52 | Nickson et al. (2016) | + | + | + | + | + | + | ? | ? | + | 7 |
| 53 | Nixon et al. (2013) | - | + | + | + | ? | + | + | + | - | 6 |
| 54 | Nusslock et al. (2011) | + | + | ? | ? | + | + | + | ? | + | 6 |
| 55 | Owashi et al. (2008) | + | ? | - | + | + | ? | - | + | - | 4 |
| 56 | Papmeyer et al. (2015) | + | + | + | + | + | + | + | + | + | 9 |
| 57 | Papmeyer et al. (2016) | + | + | + | + | + | + | + | + | + | 9 |
| 58 | Pasco et al. (2010) | + | + | + | + | + | - | + | ? | + | 7 |
| 59 | Pasquali et al. (2017) | + | + | + | + | + | ? | ? | ? | + | 6 |
| 60 | Pintor et al. (2009) | + | + | - | + | + | - | ? | + | + | 6 |
| 61 | Pintor et al. (2013) | + | + | - | ? | + | - | + | + | ? | 5 |
| 62 | Rao et al. (2009b) | ? | + | + | + | + | ? | + | + | - | 6 |
| 63 | Rao et al. (2010) | + | + | + | - | + | ? | + | - | - | 5 |
| 64 | Rao et al. (2009a) | + | + | - | + | + | + | + | ? | ? | 6 |
| 65 | Rudaz et al. (2017) | + | + | + | + | + | + | + | ? | + | 8 |
| 66 | Serra-Blasco et al. (2016) | + | + | - | + | + | + | + | + | + | 8 |
| 67 | Tsuru et al. (2013) | + | ? | - | + | + | ? | + | ? | + | 5 |
| 68 | Vinberg et al. (2014) | + | + | + | ? | + | ? | ? | - | + | 5 |
| 69 | Vrshek-Schallhorn et al. (2012) | + | + | + | + | + | + | + | + | + | 9 |
| 70 | Whalley et al. (2013) | + | + | + | - | + | + | + | ? | + | 7 |
| 71 | Whalley et al. (2015) | + | + | + | ? | + | + | ? | + | + | 7 |
| 72 | Workman et al. (2017) | + | + | + | + | + | + | + | + | + | 9 |
| 73 | Zimmerman et al. (1987) | + | + | - | + | + | ? | + | + | - | 6 |
| 74 | Zobel et al. (1999) | + | ? | - | ? | ? | - | + | ? | + | 3 |
| 75 | Zobel et al. (2001) | + | - | - | + | + | - | ? | + | + | 5 |

Interpretation of quality score: Low risk of bias or high quality score ≥7, medium risk of bias/quality score 5-6, high risk of bias or low quality score ≤4.*Supplementary Table 2.* Overview of exploratory meta-analyses of limited studies on neuroimaging, hormones and immunology.

|  | **N** | **OR/HR** | **LL** | **UL** | ***p*** | **Total n** | **Onset/ relapse/ recurrence MDD n** |
| --- | --- | --- | --- | --- | --- | --- | --- |
| **Neuroimaging: volumes** |  |  |  |  |  |  |  |
| Amygdala | 3 | 6.108 | 0.143 | 261.388 | 0.345 | 260 | 30 |
| Hippocampus | 3 | 0.660 | 0.426 | 1.022 | 0.063 | 250 | 78 |
| Frontal areas | 3 | 0.896 | 0.480 | 1.673 | 0.730 | 156 | 60 |
| **Immunology** |  |  |  |  |  |  |  |
| CRP | 4 | 1.557 | 0.870 | 2.788 | 0.136 | 15,388 | 904 |
| IL | 3 | 1.025 | 0.782 | 1.345 | 0.856 | 9430 | 698 |
| **Hormones** |  |  |  |  |  |  |  |
| Cortisol | 19 | 1.294 | 1.035 | 1.616 | 0.024* | 5492 | 494 |
| *Subgroup analyses:* |  |  |  |  | 0.035* |  |  |
| No baseline diagnosis | 6 | 1.082 | 0.938 | 1.249 | 0.280 | 3464 | 212 |
| Baseline diagnosis included | 13 | 1.919 | 1.151 | 3.200 | 0.012* | 2028 | 282 |
| Cortisol (HR) | 5 | 0.985 | 1.037 | 0.794 | 0.427 | 626 | 432 |
| Growth Hormone | 3 | 0.149 | 0.015 | 4.139 | 0.332 | 55 | 28 |
|  |  |  |  |  |  |  |  |

* indicates a significant odds ratio defined with *p*<0.05. Abbreviations: OR=odds ratio, HR=Hazard ratio; N=number of studies, n=number of participants, LL: lower limit of 95% confidence interval UL: Upper limit of 95% confidence interval, CRP=c-reactive protein, IL=interleukin.

**Supplementary References**

1. Moher D, Liberati A, Tetzlaff J, Altman DG, PRISMA Group. Preferred reporting items for systematic reviews and meta-analyses: the PRISMA statement. PLoS Med. 2009 Jul 21;6(7):e1000097.
